# Mapping human norovirus antigens during infection reveals the breadth of the humoral immune response

**DOI:** 10.1038/s41541-023-00683-1

**Published:** 2023-06-06

**Authors:** Lynn Su, Wanzhi Huang, Frederick H. Neill, Mary K. Estes, Robert L. Atmar, Timothy Palzkill

**Affiliations:** 1 Department of Pharmacology and Chemical Biology, Baylor College of Medicine, Houston, TX 77030 USA; 2 Department of Molecular Virology and Microbiology, Baylor College of Medicine, Houston, TX 77030 USA; 3 Department of Medicine, Baylor College of Medicine, Houston, TX 77030 USA

**Keywords:** Antibodies, Viral host response

## Abstract

Human noroviruses (HuNoV) are the leading cause of acute gastroenteritis worldwide. The humoral immune response plays an important role in clearing HuNoV infections and elucidating the antigenic landscape of HuNoV during an infection can shed light on antibody targets to inform vaccine design. Here, we utilized Jun-Fos-assisted phage display of a HuNoV genogroup GI.1 genomic library and deep sequencing to simultaneously map the epitopes of serum antibodies of six individuals infected with GI.1 HuNoV. We found both unique and common epitopes that were widely distributed among both nonstructural proteins and the major capsid protein. Recurring epitope profiles suggest immunodominant antibody footprints among these individuals. Analysis of sera collected longitudinally from three individuals showed the presence of existing epitopes in the pre-infection sera, suggesting these individuals had prior HuNoV infections. Nevertheless, newly recognized epitopes surfaced seven days post-infection. These new epitope signals persisted by 180 days post-infection along with the pre-infection epitopes, suggesting a persistent production of antibodies recognizing epitopes from previous and new infections. Lastly, analysis of a GII.4 genotype genomic phage display library with sera of three persons infected with GII.4 virus revealed epitopes that overlapped with those identified in GI.1 affinity selections, suggesting the presence of GI.1/GII.4 cross-reactive antibodies. The results demonstrate that genomic phage display coupled with deep sequencing can characterize HuNoV antigenic landscapes from complex polyclonal human sera to reveal the timing and breadth of the human humoral immune response to infection.

## Introduction

Human noroviruses (HuNoVs) are the leading cause of both sporadic cases and epidemic outbreaks of gastroenteritis, causing ~200,000 deaths and accounting for a global economic burden of 60 billion USD each year^1–3^. Noroviruses (NoVs) belong to the family *Caliciviridae* and are classified into 10 genogroups (GI-GX) and 49 genotypes. Five NoV genogroups (I, II, IV, VIII, and IX), which contain 38 different genotypes, are capable of infecting humans^4^. The extensive sequence diversity of HuNoVs leads to immune escape, creating a potential obstacle in the development of a broadly protective vaccine.

The NoV genome is a single-stranded, positive-sense RNA that is approximately 7.5 kilobases (kb) in length and is organized into three open reading frames (ORFs). ORF1 encodes a large polyprotein that is processed into six nonstructural proteins involved in viral replication including NS1/2 (p48), NS3 (nucleoside-triphosphatase, or NTPase), NS4 (p22), NS5 (VPg), NS6 (Protease), and NS7 (RNA-dependent RNA polymerase, or RdRp). ORF2 and ORF3 encode the major (VP1) and minor (VP2) capsid proteins, respectively. The major capsid protein VP1 is comprised of a short N-terminal arm, a shell (S) domain, and a protruding (P) domain. The S domain maintains the integrity of the NoV capsid assembly. The P domain directly binds to histo-blood group antigens (HBGAs) to infect human cells^5,6^. The P domain is further divided into P1 and P2 subdomains where the P2 subdomain is exposed on the outer surface and is highly variable in sequence, facilitating escape from an antibody response^7^. The minor capsid protein VP2 binds to a conserved motif in the VP1 S domain. Its function remains unclear, although it interacts with the viral genomic RNA in murine NoV^8^.

The humoral immune response against HuNoV infections plays a critical role in viral clearance and protection from subsequent infections^6^. Evidence suggests humoral immunity is more effective and longer lasting than T cell immunity in clearance of HuNoV infections^9^. Many monoclonal antibody (mAb) epitopes for HuNoV have been identified. A recent review summarized over 70 published studies delineating linear and conformational epitopes, mostly residing in VP1, for 307 unique mAbs^9^. Although mapping epitopes in mAbs is critical for rational vaccine design, it cannot capture the entirety of the polyclonal humoral immune response. To gain a comprehensive understanding of humoral immunity against norovirus infections, identification of epitopes in polyclonal human sera is needed. Previous work has assessed polyclonal human sera from both HuNoV-infected individuals and vaccine trial participants and determined the presence of protective immunity and cross-reactive blockade antibodies in those two cohorts^10–15^. Serum HBGA-blocking antibodies increase following HuNoV challenge and natural infections^10–12,14^. A further study examined the serological repertoire of pre- and post-immunized sera from three participants in a bivalent vaccine trial and identified a broadly protective neutralizing antibody and its cross-reactive epitope^15^. Nevertheless, the comprehensive HuNoV antigenic landscape remains incomplete. A comprehensive map of HuNoV epitopes would provide a systematic view of the humoral immune response during HuNoV infection. Such maps could address questions such as what epitopes are recognized during an HuNoV infection, if the antigenic landscape of HuNoV differs from person to person, if the landscape changes as the infection progresses, and if there are conserved and immunodominant epitopes among HuNoVs.

Phage display involves presenting peptides or proteins on the surface of the bacteriophage capsid^16^. The displayed peptides or proteins are encoded within the genome of each phage particle. Thus, large libraries of peptides can be screened for binding to target proteins by affinity selection and DNA sequencing^17–21^. By mapping the epitopes for antibodies elicited by infection, phage display coupled with Next-Generation Sequencing (NGS) can provide a high-resolution view of the epitope landscape during the humoral immune response. Recently, technologies such as VirScan, which uses a library of peptides from 206 species of virus displayed on a T7 phage platform coupled with NGS, as well as a pan-coronavirus phage display library have been utilized to profile immune responses in sera of COVID patients in order to investigate the immune responses against SARS-CoV-2^22–24^.

We have previously performed phage display affinity selections of a HuNoV genomic phage display library coupled with deep sequencing to identify epitopes of a scFv antibody and polyclonal rabbit sera against HuNoV^25^ (Fig. S1). In this new study, we aimed to characterize the repertoire of the humoral immune response against HuNoV infections by performing affinity selections coupled with deep sequencing on human sera of individuals infected with HuNoVs. Using our GI.1 Norwalk genomic phage display library and a newly constructed GII.4 HOV genomic phage display library, we profiled nine polyclonal human sera at a single timepoint after infection. In addition, we performed a longitudinal study on sera from three persons at times both pre-infection and at multiple points after infection. We identified epitopes in the nonstructural proteins NS1/2 (p48), NS3 (NTPase), NS4 (p22), NS5 (VPg), NS6 (protease), and NS7 (RdRp) as well as cross-reactive epitopes in the VP1 major capsid protein unique to GI.1 Norwalk or GII.4 HOV. We observed that the antigenic landscapes vary between individuals, but also contain common recurring epitopes. We also determined the changes in epitope profiles in individuals over a course of infection. These studies revealed the presence of epitopes in the pre-infection sera, indicating the individuals had previous HuNoV infections. New epitopes arose in nonstructural and structural proteins 7 to 30 days post-infection. Most of these epitopes, along with the epitopes detected in the pre-infection sera, persisted 180 days post-infection. Lastly, we detected epitopes common to GI.1 Norwalk and GII.4 HOV, confirming the presence of antibodies cross-reactive between genogroups. Our results show that phage display coupled with deep sequencing can successfully characterize epitopes in complex polyclonal human sera, providing a deeper understanding of the HuNoV immune response, which may aid the development of screening methods for HuNoV infections and a broadly protective vaccine.

## Results

### Phage display affinity selection and deep sequencing can identify multiple epitopes in a single sera sample

Previously, we constructed a GI.1 sheared genomic phage display library and utilized it to map the epitope of a human scFV antibody^25^ (Fig. S1). The library was also used to simultaneously map multiple epitopes from rabbit polyclonal antisera raised against GI.1 Virus-Like-Particles (VLPs)^25^. Greater than 450,000 clones were pooled to create this GI.1 genomic library, which was shown by NGS to be highly diverse with inserts displaying excellent coverage of the GI.1 open reading frames^25^.

**Fig. 1:**
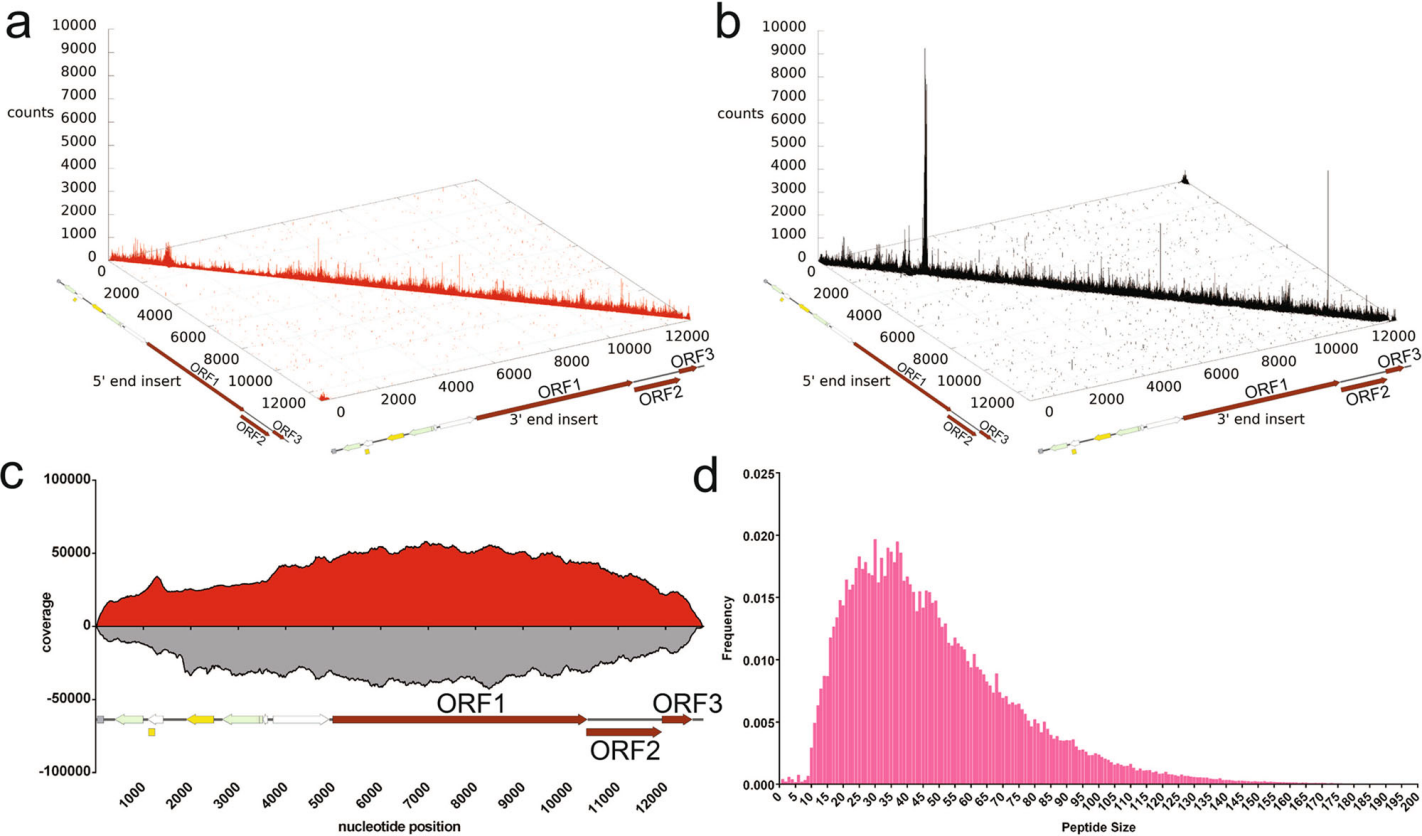
Deep sequencing analysis of the inserts present in the naive HuNoV Jun-Fos library. **a** Distribution of the forward-strand inserts (red) in the naive library mapped onto their positions in the pKS-NV68 KM plasmid. The position of the 5′ end of the insert in the pKS-NV68 KM plasmid is shown on the x-axis, while the position of the 3′ end of the insert is designated on the y-axis. The number of occurrences of the insert is defined as counts on the z-axis. **b** Distribution of reverse-strand inserts (black) in the naive library. **c** Coverage of the naive library defined by DNA sequence alignments. A total of 7,498,441 forward-strand inserts and 9,481,435 reverse-strand inserts were aligned to generate a per-nucleotide-position coverage score. The coverage score was defined as the number of occurrences of each nucleotide position in the set of DNA insert fragments aligned for the forward-strand inserts and reverse-strand inserts, respectively. The coverage scores for the forward-strand inserts of pKS-NV68 KM (red) are shown above the x-axis, while the coverage scores for the reverse-strand inserts (gray) are shown below the x-axis. **d** Distribution of peptide size in the naive library. The frequency of each unique peptide is shown on the y-axis while the peptide size is shown on the x-axis.

In this study, we again utilized the GI.1 library to map the antigenic landscape for HuNoV infection. We verified the diversity of inserts in the library by repeating NGS of the insert region of the naive library, confirming the high diversity of the library (Fig. 1a, b). The cloning of sheared DNA into the phage display plasmid results in inserts in either the forward or the reverse orientation with respect to the GI.1 genome. Note that only the forward orientation inserts will encode in-frame HuNoV peptides. We counted a total of seven million forward strand inserts and nine million reverse-strand inserts present in the library. The inserts were aligned to the reference genome to obtain a per-nucleotide coverage score^26,27^. The coverage score was calculated as the number of occurrences of each nucleotide position in the pool of both forward and reverse inserts aligned to the reference genome (Fig. 1c)^28^. The results showed that for both forward and reverse strands, multiple inserts are present for every nucleotide position in the plasmid containing the HuNoV genome, further indicating excellent coverage of the genome by inserts (Fig. 1c). The size distribution of inserts was also calculated, showing that inserts encode peptides ranging from 10 to 170 amino acids in length, with the average insert encoding 47 amino acids (Fig. 1d). The wide range of peptide lengths as well as their continuous distribution suggests that both linear and conformational epitopes are encoded by inserts in the library.

**Fig. 2:**
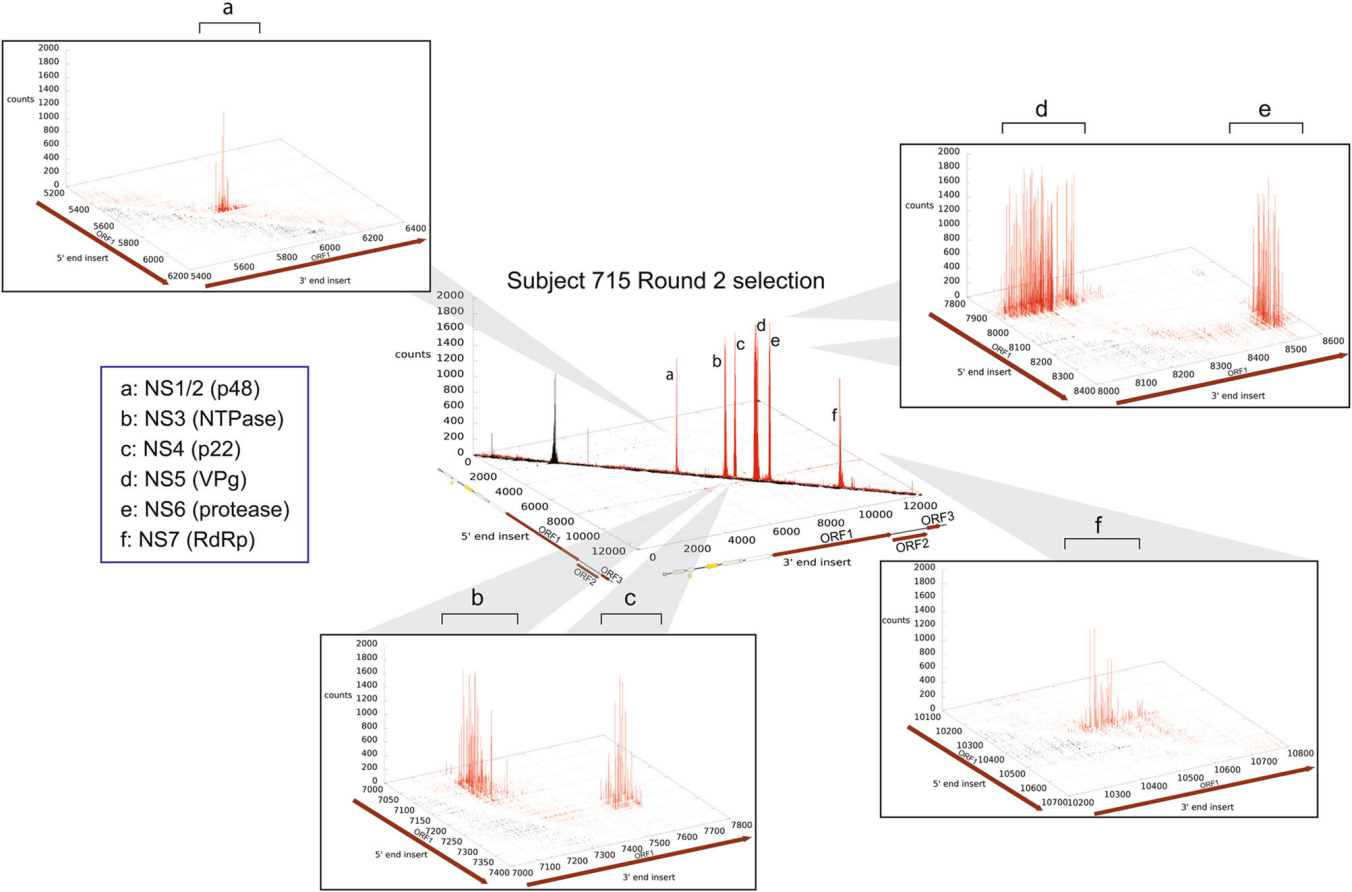
Distribution of inserts after two rounds of affinity selection with sera of subject 715 mapped onto their positions in the reference pKS-NV68 KM plasmid. The x-axis denotes the 5′ end of the inserts, y-axis the 3′ end of the inserts, and z-axis the number of occurrences. The distribution of insert clusters defines the anti-GI.1 epitopes in **a** NS1/2 (p48), **b** NS3 (NTPase), **c** NS4 (p22), **d** NS5 (VPg), **e** NS6 (protease), and **f** NS7 (RdRp).

To profile the antibody response against HuNoVs during infection, serum samples of GI.1 infected persons were used for affinity selection of the GI.1 genomic phage display library. We screened sera from six persons who were infected with GI.1 HuNoV. These sera were collected as part of a previous study^29,30^. Affinity selections were performed on sera from four persons at 14 days after infection and two persons at 30 days after infection. Affinity selections were performed by first immobilizing antibodies in the sera using protein A/G magnetic beads. The naive phage library was added to the immobilized antibodies to allow for binding. After two rounds of affinity selection, the bound phages were eluted, and the insert region was PCR amplified. Deep sequencing was performed on the PCR products to assess the distribution of inserts when aligned to the plasmid sequence that includes the HuNoV ORFs (Fig. 1). The results for individual 715 are shown in Fig. 2 and results for all six individuals are in Fig. S2.

Epitopes were identified from the deep sequencing data based on the distribution of inserts expressed as count numbers mapped to the HuNoV genome with the premise that count numbers are proportional to the extent of enrichment by antibodies in the sera^25,27^. The distribution of inserts and associated coverage scores after two rounds of affinity selection with sera from the infected individuals showed a wide range of signals that represent epitopes located in the VP1 capsid protein as well as in several nonstructural proteins (Fig. S2).

The genomic phage display library was constructed by ligating randomly sheared genomic DNA into the phage display plasmid. Because the genomic inserts can be in either orientation (forward or reverse) and any of the three reading frames, the majority of inserts in the naive library are out of frame and will not produce a HuNoV peptide. If affinity selection is enriching for HuNoV peptides recognized by antibodies in sera, the frequency of inserts encoding in-frame peptides should increase with subsequent rounds of selection, while inserts encoding out-of-frame sequences should be eliminated. As seen in Fig. S3a, approximately 20% of the inserts in the naive library encode in-frame peptides, as expected for random inserts, while by round 2 of selection, 70–90% of the inserts were found to encode in-frame peptides while inserts encoding out-of-frame sequences were largely eliminated. These results indicate that the affinity selection enriched for HuNoV sequences that bind antibodies present in sera, i.e., epitopes. We also analyzed the fraction of in-frame inserts for each count number. Figure S3b shows that the fraction of the in-frame inserts in the naive library for each insert count group stayed consistently at 20%, which is the same as what we observed for the total fraction of in-frame inserts. Also, we observed that the frequency of in-frame inserts in the libraries after selection starts to increase with inserts with a count of five. This suggests that in-frame inserts that have a count of five or above encode for peptides enriched by affinity selection, i.e., epitopes. Therefore, in-frame inserts with a count of five or above in the libraries after selection were chosen to calculate the per-nucleotide-position coverage scores, as described above for the naive library.

**Fig. 3:**
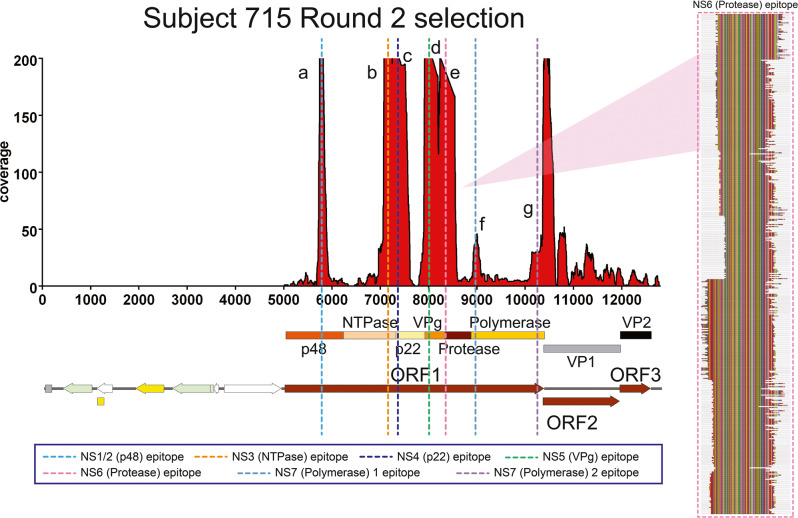
Coverage scores of the inserts present in the HuNoV Jun-Fos library and alignment of enriched peptides in NS6 (protease) after two rounds of affinity selection with sera of subject 715. The coverage of the inserts after two rounds of affinity selection with sera from subject 715 was determined by DNA sequence alignment to the plasmid containing GI.1 ORFs and is shown on the left. The per-nucleotide coverage score is shown on the y-axis while the position on the plasmid sequence is shown on the x-axis. The positions of HuNoV ORFs 1 to 3 are shown below the x-axis as a reference. Only the forward-strand inserts are shown. The alignment of enriched peptides in NS6 (protease) is shown on the right. The numbering at the top of the alignment indicates the amino acid residue positions in the nonstructural proteins. The Jalview Zappo color scheme is used, in which aliphatic/hydrophobic residues (I, L, V, A, and M) are peach, aromatic residues (F, W, and Y) are gold, positively charged residues (K, R, and H) are blue, negatively charged residues (D and E) are red, hydrophilic residues (S, T, N, and Q) are green, conformationally special residues (G and P) are magenta, and cysteine is yellow. The aligned peptide families from left to right in the coverage map defines the anti-GI.1 epitopes in NS1/2 (p48), NS3 (NTPase), NS4 (p22), NS5 (VPg), NS6 (protease), and NS7 (RdRp).

To illustrate the depth of sequencing and how the sequences composing each epitope are repeatedly enriched by the antibodies in the sera, the coverage map for study participant 715 is shown as an example in Figs. 3 and S4. The coverage map indicates that the epitopes in ORF1 are located as in-frame amino acid sequences in NS1/2 (p48), NS3 (NTPase), NS4 (p22), NS5 (VPg), NS6 (protease), and NS7 (RNA-dependent RNA polymerase, or RdRp). The coverage map also indicates the presence of epitopes in the VP1 capsid protein (not labeled in Figs. 3 and S4). The epitopes revealed by the coverage scores can be resolved at higher resolution by aligning the sequences associated with regions of high coverage scores to accurately define epitopes at single amino acid resolution (Figs. 3 and S4). For example, alignment of the sequences associated with NS4 revealed a 100 amino acid epitope. Since linear epitopes are less than 20 amino acids long^31^, the NS4 epitope is likely conformational (Figs. 3, S4, Table S1). Alignments of sequences from regions of high coverage score from this individual similarly provided a high-resolution definition of other epitopes as seen in Fig. 3 and Table S1. Taken together, these results show that the affinity selection and deep sequencing approach can identify multiple epitopes simultaneously and at high resolution from a single polyclonal serum sample.

### Antibody responses recognize similar sets of GI.1 nonstructural and structural proteins in different individuals

**Fig. 4:**
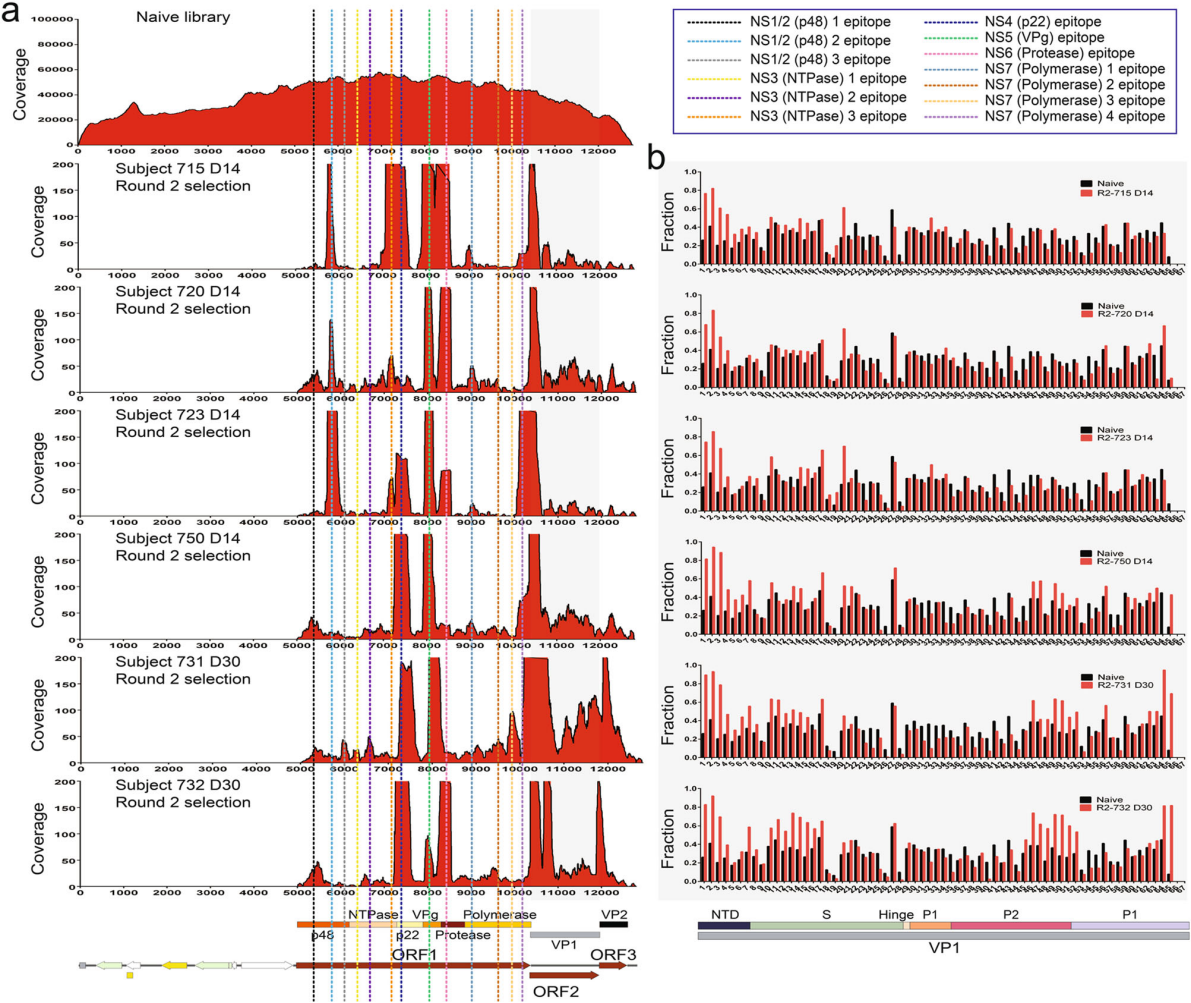
Coverage scores of the inserts present in the HuNoV Jun-Fos library versus post-infection sera before and after affinity selection. **a** The coverage of the inserts in the naive library (top) as well as the inserts after two rounds of affinity selection with sera from subjects 715, 720, 723, 731, 732, and 750 was determined by DNA sequence alignment to the plasmid containing GI.1 ORFs. The per-nucleotide coverage score is shown on the y-axis while the position on the plasmid sequence is shown on the x-axis. The positions of HuNoV ORFs 1 to 3 are shown below the x-axis as a reference. Only the forward-strand inserts are shown. **b** The fraction of in-frame inserts in ORF2 in the naive library versus in-frame inserts after two rounds of affinity selection with sera from subjects 715, 720, 723, 731, 732, and 750. An eight amino acid sliding window along GI.1 ORF2 is shown on the x-axis while the fraction of the in-frame inserts of the naive library (black bars) and libraries after two rounds of affinity selection (red bars) are shown on the y-axis.

We next compared the distribution of epitopes for antibodies elicited by HuNoV infection among the different individuals. This was accomplished by comparing the coverage score maps to detect the shared and unique epitopes among the cohort of six individuals. Epitopes were further verified by comparing the fraction of the in-frame inserts in a window that contains a potential epitope to the fraction of in-frame inserts in the naive library. If the in-frame insert frequency of the libraries after affinity selection is higher than that of the naive library in a given window, the presence of an epitope is confirmed (Fig. 4).

**Fig. 5:**
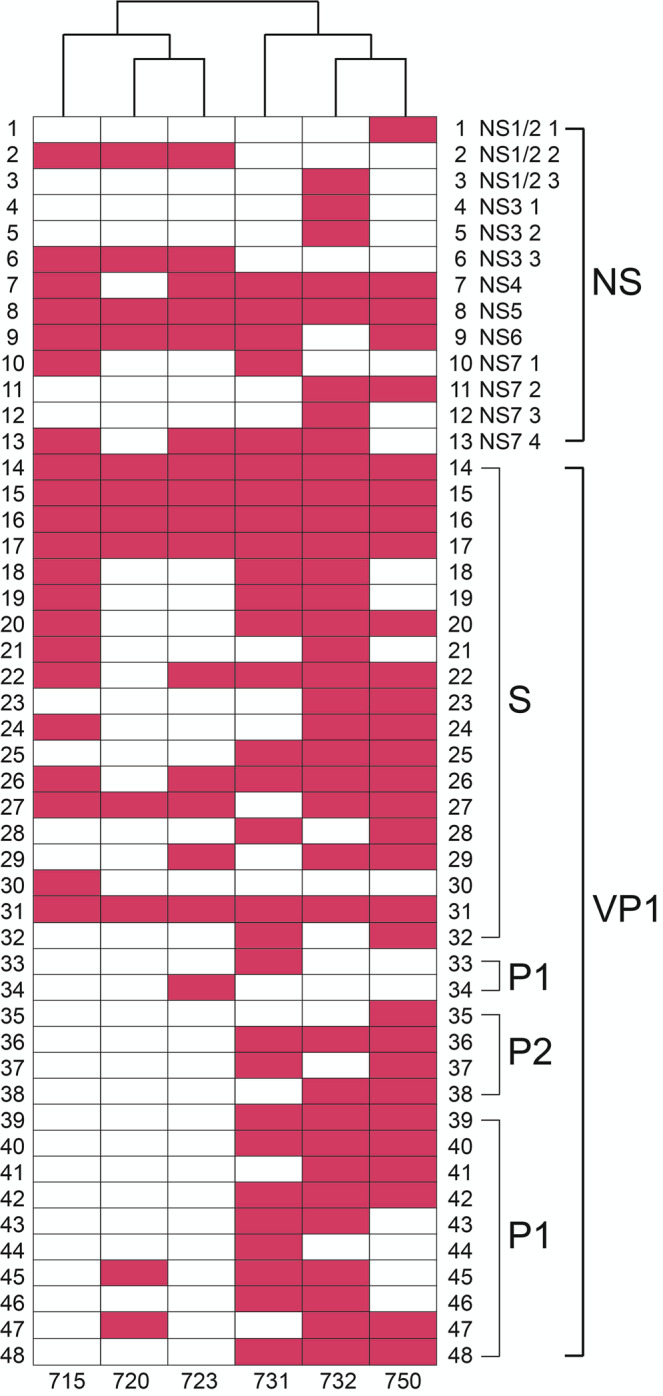
Dendrogram of GI.1 Norwalk epitope profiles. The dendrogram compares the epitope profiles shared among the six individuals. Pink blocks indicate epitopes for each individual in the specified nonstructural or structural protein domain.

We identified 13 epitopes in the nonstructural proteins. There are three prominent epitopes in NS1/2 found in multiple individuals. The alignments indicate that the epitopes map to amino acid positions 131–190, 248–282, and 315–355 of ORF1, and were found in one, two, and one individual, respectively (Figs. 4a, 5, Table S1). Another 3 prominent epitopes are in NS3, located at positions 406–448, 498–554, each found in one individual, and 697–743, found in two individuals. The 7th epitope is 100 amino acids long covering positions 759–858 of ORF1 in the N-terminal domain (NTD) of NS4. As noted, the length of this sequence suggests it is a conformational epitope. This epitope was found in five individuals. The 8th epitope is in NS5 at positions 987–1037 and was found in all six individuals. The 9th epitope is in NS6. This epitope, found in five individuals, spans 86 residues from positions 1095 to 1180. Four epitopes were identified in NS7 and are located at positions 1311–1353, 1488–1539, 1577–1653, and 1709–1780 of ORF1 with lengths of 43, 52, 77, and 72 amino acids, respectively. The first two NS7 epitopes were found in two individuals while the third epitope was found in one individual, and the last epitope was found in four individuals (Figs. 4a, 5, Table S1). The length of the sequences suggests that they are conformational epitopes.

To identify epitopes in VP1, we divided ORF2 into 67 windows that are 24 nucleotides, or eight amino acid residues long. Similar to how we defined epitopes in ORF1, we compared the frequencies of the in-frame inserts in the libraries after selection and in the naive library. If the frequency of the in-frame inserts of the selected libraries is higher than that in the naive library in a given 8-mer window, we aligned the inserts within the window to identify an epitope. Overall, we identified 35 epitopes in VP1 with 19 in the S domain, 12 in the P1 subdomain, and four in the P2 subdomain. Similar to the epitopes we found in the nonstructural proteins, most VP1 epitopes were shared among more than two individuals, with five epitopes being present in all six individuals, suggesting these epitopes are highly immunogenic (Figs. 4b, 5, Table S1).

There are 19 epitopes in the S domain, ranging in length from 12 to 35 residues. We also mapped three epitopes that were recognized by the HJT-R3-A9 scFv antibody. Based on length, the majority of these sequences appear to be linear epitopes. We identified 16 epitopes in the P domain, ranging from 8 to 38 residues in length. The majority of these sequences are fewer than 20 residues long, suggesting they are linear epitopes (Figs. 4b, 5, Table S1).

Although the majority of epitopes were found in at least two individuals, the constellation of epitopes in each individual is unique. That is, no two individuals have exactly the same set of epitopes. These results are best visualized in a dendrogram that shows similarities between individuals among the sets of recognized epitopes (Fig. 5). For example, the dendrogram shows that individuals 731, 750 and 732 have a very similar set of epitopes and thus a similar antibody response, while individuals 720 and 750 have a more divergent antibody response to infection. It is also interesting to note that there are more epitopes observed in VP1 for individuals 731 and 732, whose sera was collected at 30 days post infection, compared to the other three individuals (715, 720, and 723) who had their sera collected at 14 days post infection (Figs. 4, 5).

Taken together, the results showed that the antigenic landscape includes all of the nonstructural and structural proteins, with each person containing antibodies in their polyclonal sera to a unique collection of linear and conformational epitopes that nevertheless are widely shared among the set of individuals.

### Pre-existing epitopes and de novo epitopes associated with an adaptive response persist at 180 days post-infection

**Fig. 6:**
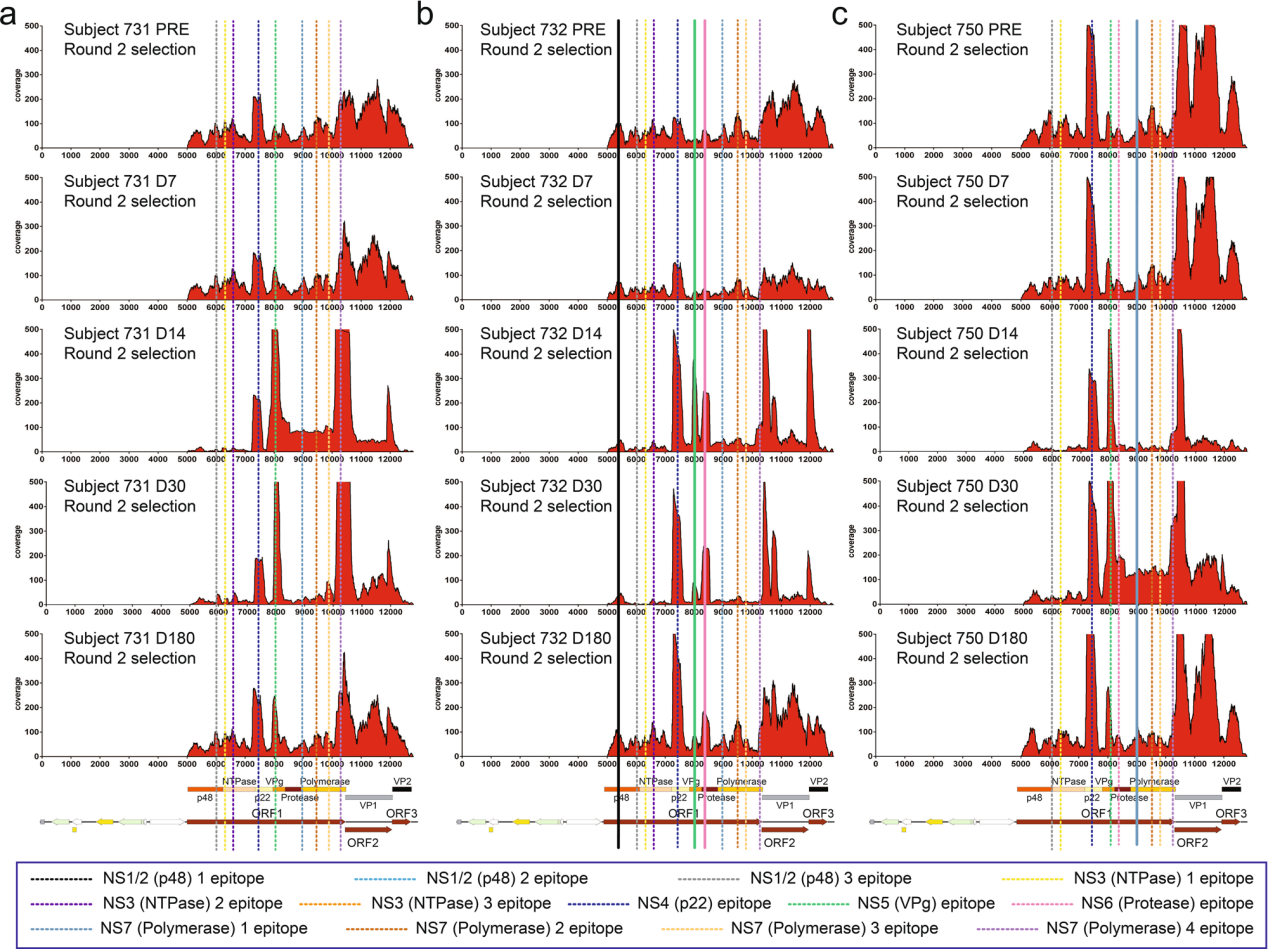
Coverage scores of the inserts present in the HuNoV library after affinity selection. The coverage of the inserts after two rounds of affinity selection with sera collected at different timepoints from subjects 731, 732, and 750 was determined by DNA sequence alignment to the plasmid containing GI.1 ORFs. The per-nucleotide coverage score is shown on the y-axis while the position on the plasmid sequence is shown on the x-axis. The positions of HuNoV ORFs 1 to 3 are shown below the x-axis as a reference. Only the forward-strand inserts are shown. **a** The coverage of inserts after two rounds of affinity selection with subject 731 sera before challenge, and 7, 14, 30, and 180 days after challenge. **b** The coverage of inserts after two rounds of affinity selection with subject 732 sera before challenge, and 7, 14, 30, and 180 days after challenge. **c** The coverage of inserts after two rounds of affinity selection with subject 750 sera before challenge, and 7, 14, 30, and 180 days after challenge. Dotted lines represent pre-existing epitopes that persist from pre-infection sera through 180 days after infection while solid lines represent epitopes that emerge post-infection.

Previous studies have estimated that protective immunity against HuNoV infections ranges from 4.1 to 8.7 years^32–34^. However, the antigenic landscape associated with humoral immunity throughout an infection is unclear. Therefore, we performed a longitudinal study of the epitope landscape associated with the antibody response over the course of HuNoV infections in multiple individuals. For this purpose, we surveyed sera collected over a span of 180 days, including five different timepoints for study subjects 731, 732, and 750. The five timepoints included pre-infection, 7, 14, 30, and 180 days post-infection. The insert count distribution and coverage scores obtained after two rounds of affinity selection for each timepoint revealed epitopes along the HuNoV ORFs. For clarity, only the epitopes in the nonstructural proteins were labeled. Each of the study participants had antibodies recognizing defined epitopes for HuNoV in the pre-infection sera, indicating they had previously been infected with HuNoV (Figs. 6, S5). These pre-existing epitopes were verified by confirming that the fraction of in-frame inserts was greater in the selected libraries than that in the naive library. All the epitopes identified in individual 731 were pre-existing and the majority of them persisted throughout the entire course of infection. There were three epitopes that emerged post-infection in subject 732, which are the first epitopes in NS1/2, NS5, and NS6, and one epitope that appeared post-infection in subject 750, which is the first epitope in NS7 (Figs. 6, S5). The remainder of the epitopes in subjects 732 and 750 were pre-existing.

Among the epitopes that arose post-infection, the epitope in NS5 showed increased enrichment for all three individuals and reached the highest coverage scores at 14 and 30 days for subjects 731 and 750 and at 14 days for subject 732. The epitopes in NS4 and NS6 for subject 732 also showed higher enrichment at 14 days until 180 days post-infection. This indicates that immune responses targeting combinations of the epitopes in NS4, NS5, and NS6 were induced at the onset of the infection for different individuals. Finally, by 180 days after infection, the insert counts and coverage scores have reverted to the baseline epitope landscape observed before HuNoV challenge for the majority of epitopes. Specifically, antibodies that were induced post-infection as part of the adaptive response also remained at day 180 (i.e., NS4 and NS6 for subject 732). Overall, these results show that the epitope landscape differs between individuals and that the antibodies present during a HuNoV infection are a combination of pre-existing antibodies presumably arising from an earlier infection as well as new antibodies induced by the HuNoV challenge. It is noteworthy that these pre-existing, persistent antibodies apparently did not provide protection from infection in that all of these individuals were infected in the presence of the antibodies.

### Affinity selections versus sera from GII.4 infected individuals reveal epitopes common to both GI.1 Norwalk and GII.4 HOV genotypes

**Fig. 7:**
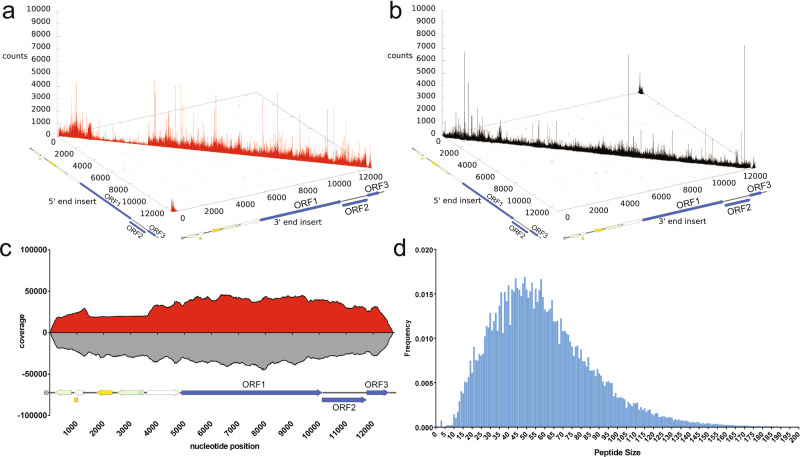
Deep sequencing analysis of the inserts present in the naive GII.4 HOV Jun-Fos library. **a** Distribution of the forward-strand inserts (red) in the naive library mapped onto their positions in the plasmid containing the GII.4 HOV ORFs. The position of the 5′ end of the insert in the plasmid is shown on the x-axis, while the position of the 3′ end of the insert is designated on the y-axis. The number of occurrences of the insert is defined as counts on the z-axis. **b** Distribution of reverse-strand inserts (black) in the naive library. **c** Coverage of the naive library defined by DNA sequence alignments. A total of 8,364,766 forward-strand inserts and 5,535,252 reverse-strand inserts were aligned to generate a per-nucleotide-position coverage score. The coverage score was defined as the number of occurrences of each nucleotide position in the set of DNA insert fragments aligned for the forward-strand inserts and reverse-strand inserts, respectively. The coverage scores for the forward-strand inserts of the plasmid (red) are shown above the x-axis, while the coverage scores for the reverse-strand inserts (gray) are shown below the x-axis. **d** Distribution of peptide size in the naive library. The frequency of each unique peptide is shown on the y-axis while the peptide size is shown on the x-axis.

Noroviruses are genetically diverse and are classified into 10 different genogroups broadly based on sequence identity and are further subdivided into 49 genotypes^4^. Ideally, a norovirus vaccine would target epitopes conserved across genogroups and genotypes. GII.4 HuNoVs have been responsible for the majority of outbreaks over the past 15 years worldwide^35–37^. They also induce more severe symptoms that require intense medical care in children^38^. To understand more about the cross-reactivity of the immune response against HuNoVs as well as the antigenic landscape of GII.4 norovirus, we generated a GII.4 HOV sheared genomic phage display library, using the same methods as described for the GI.1 library. Deep sequencing of the GII.4 HOV naive library revealed a dense distribution of both the forward and reverse inserts along the plasmid sequence with the GII.4 HOV ORFs (Fig. 7a, b). Approximately eight million forward inserts and five million reverse inserts were aligned to obtain a per-nucleotide position coverage score (Fig. 7c). The results revealed excellent coverage at every nucleotide position of the plasmid containing the GII.4 HOV genome that was used to construct the library. The distribution of peptide sizes encoded by the inserts range from approximately 10 to 180 amino acids with an average size of 58 amino acids (Fig. 7d). Similar to the GI.1 library, the GII.4 HOV library had a continuous distribution of peptide sizes, indicating that the insert sizes are very diverse.

We performed affinity selections using the GII.4 HOV library against sera from three individuals with high serum anti-GII.4 Sydney 2012 antibody levels, one of whom (BCM16-1) had a recent GII.4 Sydney 2012 virus infection. We additionally performed affinity selection using the GII.4 HOV library against the scFv HJT-R3-A9 antibody and rabbit anti-GI.1 VLP antisera, both of which bind GI.1 VP1. The frequency of inserts encoding in-frame HuNoV peptides in the GII.4 HOV naive library and after each round of affinity selection were assessed. Figure S6a shows that, similar to the progression of affinity selections from rounds one to two using the GI.1 library, affinity selection enriched for HuNoV sequences that bind to serum antibodies. The fraction of in-frame inserts for each count number, as shown in Figure S6b, also began to increase at inserts with a count of five, though not as apparent as the results for GI.1. The inserts with a count of five or above were used to calculate the per-nucleotide-position coverage scores.

**Fig. 8:**
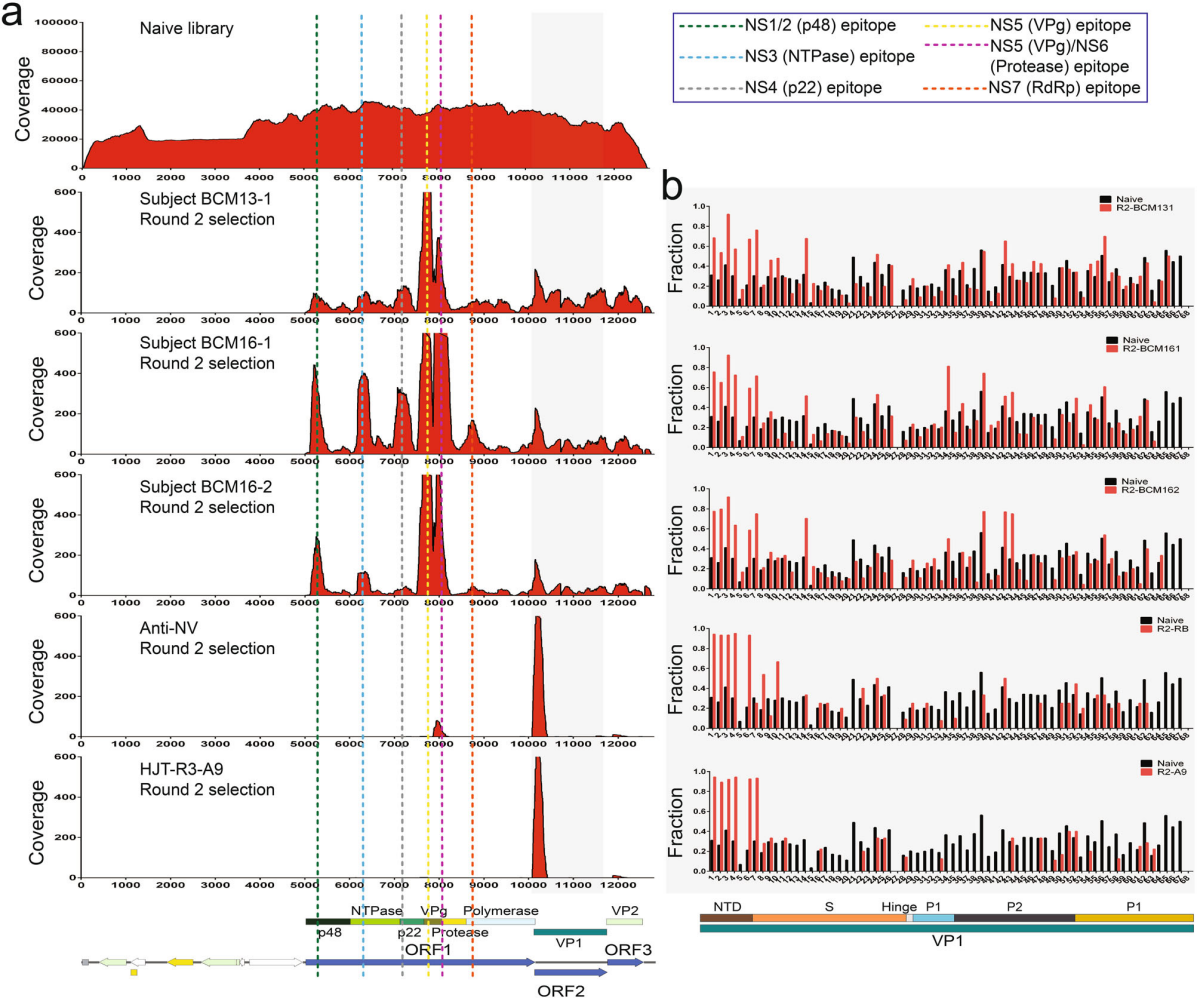
Coverage of the inserts present in the GII.4 HOV Jun-Fos library versus GII.4 antisera before and after affinity selection. **a** The coverage of the inserts in the naive library (top) as well as the inserts after two rounds of affinity selection with sera from subjects BCM16-1 sera, BCM13-1 sera, BCM16-2 sera, anti-NV rabbit polyclonal antibodies, and HJT-R3-A9 scFv antibody determined by DNA sequence alignment to the plasmid containing the GII.4 HOV ORFs. The per-nucleotide coverage score is shown on the y-axis while the position on the plasmid is shown on the x-axis. The positions of GII.4 HOV ORFs 1 to 3 are shown below the x-axis as a reference. Only the forward-strand inserts are shown. **b** The fraction of in-frame inserts in ORF2 in the naive library versus in-frame inserts after two rounds of affinity selection with sera from subjects BCM16-1, BCM13-1, BCM16-2, anti-NV rabbit polyclonal antibodies, and HJT-R3-A9 scFv antibody in GII.4 HOV ORF2. An eight amino acid sliding window along GI.1 ORF2 is shown on the x-axis while the fraction of the in-frame inserts of the naive library (black bars) and libraries after two rounds of affinity selection (red bars) are shown on the y-axis.

The insert count and coverage maps generated after deep sequencing of the GII.4 HOV library selections revealed epitopes that are located in NS1/2 (p48), NS3 (NTPase), NS4 (p22), NS5 (VPg), NS6 (protease), and the NS7 (RdRp) (Figs. 8, S7, Table S2). Epitopes mapping to NS1/2 from sera of all three individuals share an epitope from residues 68 to 113 of ORF1. The epitope in NS3 was also shared with all three persons and is 81 residues long (395–475), suggesting it is a conformational epitope. The epitope in NS4 also appeared in all three individuals with a sequence of 78 residues (702–779). The epitope in NS5 has two residues that overlap the C-terminus of NS4 and appeared in all three individuals. This epitope has a common sequence of 69 residues (872–942), suggesting it is a conformational epitope. Finally, the epitope in NS6 overlaps NS5 and NS6 is also shared among all three individuals and is located from position 982 to 1029 of ORF1. The last epitope was only present in subject BCM16-1 and is in NS7. This epitope is 64 residues long (1220–1283), again suggesting a conformational epitope (Figs. 8, S7, Table S2).

Affinity selection of the GII.4 HOV library against these targets also showed enrichment of sequences from VP1. As with the GI.1 experiment, we divided ORF2 into 68 windows that are 24 nucleotides, or eight residues long, and compared the frequency of the in-frame inserts of the selected libraries and the naive library in each window. The in-frame inserts with a higher frequency after selection than that in the naive library were aligned to identify epitopes. We identified a total of 18 epitopes in VP1 with 10 in the S domain, four in the P1 subdomain, and four in the P2 subdomain. Six individuals had a unique epitope. Among the 30 individuals that had more than two epitopes, five epitopes in the NTD of S domain were present in all six individuals, suggesting these five epitopes are highly immunogenic. (Fig. 8, Table S2). The epitope profiles of the sera from the three individuals as well as the A9 antibody, and anti-NV rabbit sera are shown in the dendrogram, which shows that the immune profiles between the A9 antibody and the anti-NV sera are the most similar while the profiles among the sera are more closely related, with BCM16-1 and BCM16-2 having the most similar epitope profile (Fig. S8).

Overall, fewer epitopes were identified using the GII.4 HOV library against the sera from GII.4 Sydney infected individuals than that observed in the selections of the GI.1 library against the sera of GI.1 infected individuals. This is likely due to the use of the GII.4 HOV library against sera from individuals infected with a different GII.4 genotype. In addition, the GII.4 sera were from natural infections and the timeline between infection and sera collection is unclear. The timing of sera collection is expected to impact the epitopes observed.

Since the individuals were not infected with GII.4 HOV 2002 and the A9 antibody and anti-rabbit VLP sera were shown to identify GI.1 and GII.4 epitopes, the epitopes identified from affinity selection of the GII.4 HOV library versus these targets would be for cross-reactive antibodies that recognize both GI.1 and GII.4. To identify cross-reactive epitopes and determine the level of sequence conservation within the epitopes, we aligned the sequences of the GI.1 and GII.4 HOV epitopes (Fig. S9a) and calculated the percent identity (PI) between the GI.1 and GII.4 HOV epitope sequences. The alignments revealed that NS7 epitope has the highest PI value, 81.4%, among all the epitopes (Fig. S9a), indicating that this epitope is the most conserved between genogroups. The epitope with the lowest PI value (27.5%) is that in NS1/2. Within VP1, the epitope with the highest PI value (79.5%) is in the S domain, or the 11th epitope in Fig. S9a. The epitope with the lowest PI value (31.6%) is in the NTD of the S domain, or the 7th epitope in S9a. The high levels of conservation for most of the cross-reactive epitopes in the nonstructural and structural proteins suggest possible integration into a broadly protective vaccine if they exhibit HBGA-blocking properties. In addition, they could find use as broadly reactive diagnostic tools to detect norovirus infections (Fig. S9). Finally, we searched the sequences of the norovirus epitopes identified here using protein BLAST and did not identify high sequence similarity matches to viruses other than noroviruses. Thus, the antibodies binding the epitopes we identified are likely specific for noroviruses.

Taken together, the results for the GI.1 and GII.4 HOV post-infection sera, anti-NV sera, and scFv HJT-R3-A9 antibody showed that phage display affinity selections coupled with deep sequencing can be used on complex polyclonal human sera to simultaneously identify multiple epitopes. Our data identified 48 GI.1 and 24 GII.4 HOV epitopes in the nonstructural and structural proteins. Similar epitope profiles were identified among infected individuals, although unique epitopes were also present. We also observed that epitopes change over the course of infections, illustrating the dynamic nature of the adaptive immune response. Lastly, we observed antibodies that are cross-reactive across GI.1 and GII.4 HOV. The 10 epitopes recognized by the cross-reactive antibodies have the potential to be incorporated into a vaccine or diagnostic tool for the detection of norovirus infections.

## Discussion

Here, we showed that Jun-Fos-assisted phage display coupled with deep sequencing can simultaneously map multiple epitopes from complex polyclonal human sera. We utilized this approach with GI.1 and GII.4 HOV genomic libraries to determine the epitope landscape for nine complex polyclonal human sera of HuNoV-infected individuals and a longitudinal collection of 15 sera from five timepoints of three infected individuals. Our data revealed multiple GI.1 epitopes in each individual, including epitopes in both nonstructural and structural proteins. Using the longitudinal sera samples, we observed that all individuals had pre-existing epitopes as well as new epitopes due to the adaptive immune response resulting from the challenge GI.1 infection. In addition, the mapping of epitopes from both GI.1 and GII.4 sera allowed the identification of cross-reactive epitopes. Altogether, the results provide insights into the humoral immune responses to HuNoV infection.

Virus-Like-Particles (VLPs), which are composed of the major structural protein VP1, are highly immunogenic and are vaccine candidates due to their well-studied immunogenicity and epitope profiles^39^. In contrast, the immunogenicity and epitope landscapes of HuNoV nonstructural proteins have not been as well characterized. However, it has been shown that an expression clone located in the C-terminus of NS3 (NTPase) and N-terminus of NS4 (p22) isolated from immunological screening was reactive with GI.1 norovirus post-infection sera^40^. Another report showed that individuals infected with a GI.1 norovirus had a seroresponse to the nonstructural fusion protein VPR, which is composed of VPg, protease, and RdRp^41^. Further, a recent study that surveyed humoral immune responses against HuNoV in children across Europe reported antigenicity in NS1/2 (p48) and NS4 (p22)^42^. Our work greatly expands the understanding of the immunogenicity of nonstructural proteins through the identification of epitopes in multiple nonstructural proteins, including those listed above. Specifically, we identified 13 GI.1 epitopes and six GII.4 HOV epitopes in nonstructural proteins.

The location of epitopes on protein structures provides clues as to whether antibodies binding these epitopes possess neutralizing activities. X-ray structures of NS6 (protease) and NS7 (RdRp)^7,43–46^ and Nuclear Magnetic Resonance (NMR) analysis of murine norovirus VPg and a homology model of HuNoV VPg^47,48^ are available. HuNoV NS5 (VPg) covalently binds to 5′ end of the viral RNA genome and participates in the initiation of viral RNA replication. The cross-reactive NS5 epitopes for GI.1 (984–1037) and GII.4 HOV (872–942) (Fig. S9) map to the structured helical core of VPg and include a critical tyrosine residue (Y992 in GI.1 and Y902 in GII.4 HOV), suggesting antibodies that bind to these epitopes can directly inhibit the viral RNA replication step^47^.

**Fig. 9:**
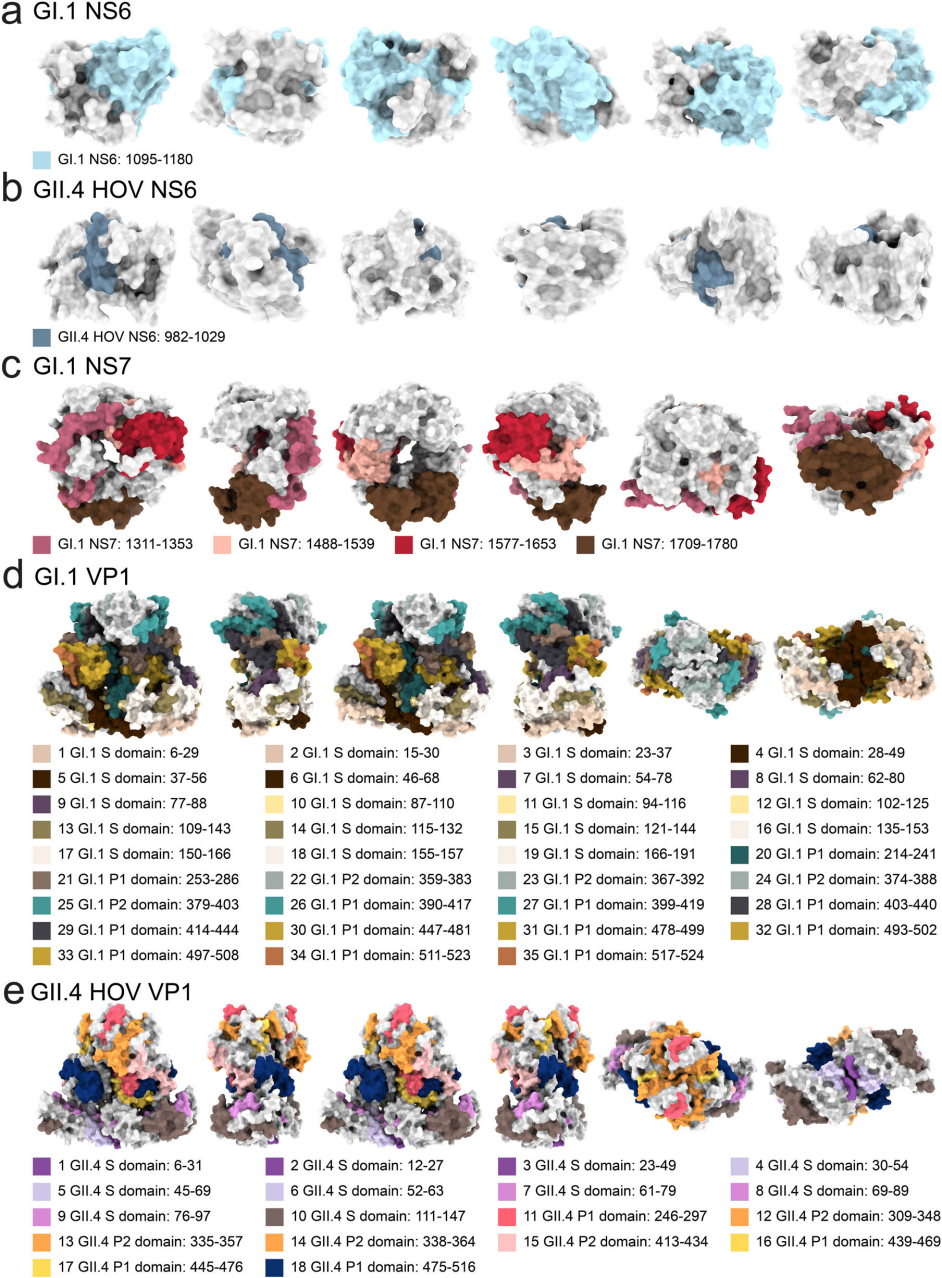
Location of epitopes on the GI.1 Norwalk and GII.4 HOV NS6 (protease), GI.1 NS7 (RdRp), GI.1 VP1, and GII.4 HOV VP1 structures. Location of epitopes on the GI.1 Norwalk and GII.4 HOV NS6 (protease), GI.1 NS7 (RdRp), GI.1 VP1, and GII.4 HOV VP1 structures. From left to right, four surface diagrams that are rotated 90˚ counterclockwise on the y-axis and two surface diagrams rotated 90˚ counterclockwise on the x-axis are shown for the **a** GI.1 NS6 structure (PDB ID: 2FYQ), **b** GII.4 HOV NS6 structure (PDB ID: 6NIR), **c** GI.1 NS7 structure (PDB ID: 4NRT), and **d** GI.1 VP1 (PDB ID: 1IHM), **e** GII.4 VP1 (PDB ID: 7MRY) with the location of epitopes.

We also identified epitopes in HuNoV NS6 (protease) in both GI.1 and GII.4 HOV. The NS6 epitope detected from GI.1 sera (1095–1180) maps to the cleft of the protease, which includes key catalytic residues in the active site (H1130 and E1154) (Fig. 9a)^43,49^. Antibodies targeting this epitope therefore could inhibit the catalytic activity of NS6. The GII.4 HOV NS6 epitope (Fig. 9b) maps to the first 21 residues and does not include the active site of the protease^43^.

We identified four epitopes in the GI.1 NS7 protein (RdRp), which is responsible for the replication of the viral RNA. Two of the four GI.1 NS7 epitopes overlap the active site of NS7 in the palm region (D1522, D1623, D1624)^50,51^ and another epitope includes residues in the thumb region (I1721, S1722, E1726, W1764, M1765) that interact with the C-terminal tail in the active site cleft during RNA replication^52^ (Fig. 9c). Therefore, antibodies that bind to these GI.1 polymerase epitope could inhibit RNA synthesis.

We identified 35 epitopes in the GI.1 major capsid protein VP1, with 19 in the S domain, 12 in the P1 subdomain, and four in the P2 subdomain (Fig. 9d). Many epitopes aligned with the epitopes noted by van Loben Sels and Green^9^. The first five epitopes, which span residues 6 to 56, are in the NTD of the S domain and are highly immunogenic as they appeared in all six study subjects. There were 15 previously identified GI.1 mAbs that also recognize sites in the 1–43 region of the NTD of the S domain^53^. The 14 other epitopes in the S domain cover most of the eight β strands B to I^7^. These have not been reported as neutralizing epitopes. The P2 subdomain is the most antigenic region and contains the binding site for human HBGA during viral infections^54^. We identified four epitopes in P2. These epitopes were all shown to be recognized by HBGA-blocking, or neutralizing antibodies (Fig. 9d)^5,55^. A majority of the conformational epitopes bound by neutralizing antibodies such as 5I2 and several nanobodies (Nano-7, Nano-62, and Nano-94) are also contained in GI.1 22nd to 25th epitopes (Fig. 9d)^56–58^.

Our GII.4 HOV results indicated 18 epitopes in VP1, with 10 in the S domain, four in the P1 subdomain, and four in the P2 subdomain, with most of the epitopes identified also discussed by van Loben Sels and Green^9^. Many residues in the epitopes identified in the P1 and P2 subdomains are also conserved across the GII genotypes. For example, a continuous sequence in the 4th and 5th epitope in the GII.4 S domain shown in Fig. 9e is highly conserved across GII.4, GII.7, and GII.8 and is recognized by MAb N2C3^59^. The 11th P2 subdomain epitope in Fig. S9e also contains several residues (A294, G295, S296, N298) that are highly conserved across many GII.4 genotypes and are a part of epitope A, which was previously identified as an immunodominant blockade epitope^60–70^. The 12th epitope, also located in P2 subdomain, contains V327, which is a highly conserved residue in another blockade epitope (epitope F) recognized by GII.4F, a human monoclonal antibody generated after acute infection^63,65,71–73^. The 12th epitope also contains a part of the blockade epitope recognized by a GII-specific monoclonal antibody NORO-320^74,75^. The 11th and 12th epitopes are located at the top of VP1 (Fig. 9e). Our 16th and 17th epitopes in the P1 subdomain contain residues within an HBGA-blocking epitope that is recognized by the monoclonal antibody 3C3G3 as well as the cross-reactive monoclonal antibodies NV23, NS22, and F120^76–79^. The 16th and 17th epitopes are located on the side of the VP1 (Fig. 9e). Lastly, our 13th and 18th epitopes contain residues in two subdominant blockade epitopes, C and I, which were shown to have modest blockade and neutralization titers^70^. The epitopes in the P1 and P2 subdomains that we identified in GI.1 and GII.4 HOV VP1 include many highly conserved, cross-reactive, and key residues targeted by HBGA-blocking antibodies. Antibodies that inhibit HBGA-blocking have been shown to correlate with clinical GI.1 and GII.4 neutralization and protection against HuNoV infections^10,12,80^. Some antigenic epitopes were also confirmed to be strongly neutralizing (i.e., epitope A)^70^. The GI.1 and GII.4 HOV epitopes that contain HBGA-blocking residues would therefore be candidates for incorporation into protective vaccines.

Our results clearly show that HuNoV infection elicits antibodies that bind to nonstructural proteins. However, nonstructural proteins are made inside of virus-infected cells, and it is not clear how these proteins would be available for interaction with B cell receptors (BCRs) to generate an adaptive antibody response. Cell lysis following infection could release nonstructural proteins for binding to lymphocyte receptors, resulting in antibody production. Apoptosis of the infected cell is a possible mechanism for cell lysis to release nonstructural proteins. Lee et al. demonstrated that the murine norovirus NS1 and a form of HuNoV NS1 are secreted^81^. They speculated that NS1 secretion is coupled with apoptosis since it is facilitated by caspase-3 cleavage. Another study found that when only murine norovirus ORF1 polyproteins are expressed in a eukaryotic cell system, apoptosis is induced through caspase-9 activation^82^.

An additional question is whether antibodies to nonstructural proteins could mediate protection from infection. As noted above, several of the identified epitopes map to the active site or functional regions of nonstructural proteins and could block activity. However, it is not clear how antibodies would gain access to the nonstructural proteins since the proteins function within infected cells. Another path by which the antibodies could enhance protection is through their Fc effector functions. Antibodies elicited from nonstructural proteins can activate Fc-mediated effector functions that are used to clear virus-infected cells, including antibody-dependent cellular cytotoxicity (ADCC) or antibody-dependent cellular phagocytosis (ADCP)^83^.

Although there are limited reports of epitopes in HuNoV nonstructural proteins, several previous studies have demonstrated that nonstructural proteins of other viruses are rich with epitopes. For instance, a study on COVID-19 patients identified IgG epitopes in all SARS-CoV-2 nonstructural and accessory proteins^84^. Interestingly, epitopes correlated with increased patient survival were focused in the NSP3 and NSP5 proteases, whereas IgG epitopes that reside in the Spike, Nucleocapsid, and ORF3a accessory proteins were associated with increased mortality^84^. In addition, the discovery of cross-reactive anti-NS1 epitopes in Dengue and Zika viruses led to the suggestion that NS1 is a viable vaccine candidate and led to passive protection in animal models^85–88^. Whether epitopes from HuNoV nonstructural proteins provide protection to infection is unknown. However, our results coupled with the findings with other viruses suggest that nonstructural proteins that induce cross-reactive antibodies are candidates for further investigation and possible use as a screening tool to detect viral infections.

Our findings also demonstrated the dynamic nature of the longitudinal antibody response over the course of an HuNoV infection. Epitopes identified in the pre-infection sera were present when de novo epitopes were induced post-infection for all study subjects. One explanation for this observation is that the challenge-induced antibodies were against both the GI.1 Norwalk strain and strains that previously infected the individuals. These patterns are similar to “back-boosting” observed in influenza virus immunology, where immune responses are induced against epitopes in the currently infecting strain as well as the previous strains if the epitopes have high homology^89^. This heterogenous immunity where increased titers of antibodies are generated against conserved epitopes shared between the current and previous strains was also recently observed in SARS-CoV-2 infections^90,91^.

We utilized a GI.1 and a GII.4 HOV genomic phage display library and performed affinity selections against nine unique human sera, 12 sera from different timepoints of three HuNoV infections, a rabbit antiserum, and a monoclonal scFV antibody. Deep sequencing of inserts from the affinity selections identified epitopes in many nonstructural proteins. A role for these antibodies binding these epitopes, if any, for immunity is unclear and worthy of future study. Furthermore, our work revealed extensive longitudinal changes of epitope persistence during HuNoV infections. Finally, we identified cross-reactive epitopes in sera from both GI.1 and GII.4 infected individuals, suggesting cross-genogroup antibodies are elicited during infection. Future work with sera from a more extensive cohort will provide further understanding of epitopes and thus antibody dynamics with respect to immunity against HuNoV infections.

## Methods

### Clinical samples

A total of 21 serum samples were included in this study. Six persons who were infected during a GI.1 human experimental infection study provided 18 samples^30^, and three persons with naturally-acquired antibodies to GII.4 NoV provided individual samples. Samples from subjects 715, 720, and 723 in the GI.1 study were collected two weeks after challenge; samples from 731, 732, and 750 were collected before challenge and 1, 2, 4, and ~26 weeks after challenge. Subjects 720, 723, 731, and 732 were diagnosed with gastroenteritis after the challenge while subjects 715 and 750 were not^30^. Serum samples from BCM13-1 were collected in 2013 while BCM16-1 and BCM16-2 were collected in September and May of 2016, respectively. The study was approved by the Baylor College of Medicine Institutional Review Board.

### Library construction

The GI.1 pTP663 Jun-Fos phage display library containing inserts from the sheared pKS-NV68 KM plasmid, which encodes GI.1 ORF1 to ORF3, was previously constructed^25^. A total of 12.8 μg pKS-NV68 plasmid was sheared using a Covaris S2 focused ultrasonicator to a size range of 100 to 500 bp. The sheared DNA was purified using a Qiagen PCR purification column and eluted in 50 μg of 10 mM Tris-Cl (pH 8.5) buffer, resulting in 56 ng/μl and 2.8 μg as the final concentration and amount of DNA, respectively. The ends of the resulting 2.8 μg DNA fragments were blunted and phosphorylated in buffer consisting of 50 mM Tris-Cl, 10 mM MgCl_2_, 1 mM ATP, 10 mM dithiothreitol (DTT), 0.4 mM deoxynucleoside triphosphates (dNTPs), and 15 units T4 DNA polymerase (New England Biolabs [NEB]), and 50 units T4 polynucleotide kinase (NEB), and 5 units Klenow fragment DNA polymerase, in a 100 μg total volume. The reaction mixture was incubated at 20 ˚C for 30 min, purified using a Qiagen PCR purification column and eluted in 10 mM Tris-Cl (pH 8.5) buffer.

To prepare the DNA fragments for ligation with the pTP663 Jun-Fos phage display plasmid via A-T cloning, deoxyadenosine was added to the end-repaired DNA fragments mentioned above in a 50 μl reaction mixture containing 50 mM NaCl, 10 mM Tris-Cl, 10 mM MgCl_2_, 1 mM DTT, and 0.2 mM dATP with 15 units of Klenow DNA polymerase exonuclease (NEB). The reaction mixture was incubated for 30 min at 37 ˚C, purified and eluted in 10 mM Tris-Cl (pH 8.5) buffer.

After growth in a *dam*-negative strain of *E. coli*, the pTP663 Jun-Fos phage display plasmid was prepared for cloning by purifying plasmid DNA using a Qiagen miniprep kit. A total of 9 μg of plasmid DNA was digested with XbaI in 1X NEB CutSmart buffer at 37 ˚C for 2 h in a volume of 200 μl followed by the inactivation of XbaI at 65 ˚C for 10 min. The DNA ends of the pTP663 Jun-Fos phage display plasmids were made blunt by addition of 4 μl of 10 mM dNTPs and 2 μl of Klenow DNA polymerase (NEB) and incubation at 25 ˚C for 30 min, followed by incubation at 70 ˚C for 10 min. The reaction mixture was purified and eluted in 10 mM Tris-Cl (pH 8.5) buffer. Dideoxythymidine was added to the purified DNA in a 100 μl reaction mixture with 1X terminal deoxynucleotidyl transferase (TdT) buffer (NEB) with 0.25 mM CoCl_2_, 0.2 mM ddTTP, and 40 units of TdT (NEB). The reaction mixture containing T-tailed plasmids was incubated for 1 h at 37 ˚C followed by 10 min at 70 ˚C and separated by agarose gel electrophoresis. The 5300 bp pTP663 band was extracted with a Qiagen gel purification kit and eluted in 80 μl of 10 mM Tris-Cl (pH 8.5) buffer, resulting in a final concentration of 6.7 ng/μl of pTP663 DNA.

The sheared and A-Tailed DNA of 100 to 500 bp (1.45 μg) was ligated with the T-tailed pTP663 vector DNA (0.13 μg) in a 250 μl reaction volume of 50 mM Tris-Cl, 10 mM MgCl_2_, 1 mM ATP, 10 mM DTT buffer at 16 ˚C for 12 h. The reaction was then extracted with phenol-chloroform-isoamyl ethanol, precipitated in 2 volumes of 100% ethanol at −80 ˚C for 1 h, and centrifuged at 13,000 rpm for 15 min. The DNA pellet was dried and resuspended in TE buffer (10 mM Tris-Cl, 1 mM EDTA [pH 8.0]), followed by electroporation of 3 μl DNA into 50 μl *E. coli* XL1-Blue electrocompetent cells. Transformed cells were selected with 100 μg/ml ampicillin on agar plates. The library size was estimated by the colony count number and the percentage of clones with inserts was determined via deep sequencing of the individual clones. The remaining 445,000 colonies were pooled by mixing LB medium with 100 μg/ml ampicillin with the colonies on the agar plates into a slurry.

Phage production from the pooled library cells was carried out by adding 0.1 ml of the pooled library cells to 30 ml of 2YT medium with 100 μg/ml ampicillin. The culture was incubated at 37 ˚C while shaking for approximately 3 h until the optical density at 600 nm (OD_600_) reached 0.6. KM13 helper phage^92^ was added to 1 × 10^10^ phages/ml and the cultures were incubated for 30 min at 37 ˚C without shaking and subsequently for 20 h at 30 ˚C with shaking. The removal of *E. coli* cells was done by centrifugation and the precipitation of phages was carried out by adding 1/5 volume of 20% polyethylene glycol 6000 (PEG 6000), 2.5 M NaCl. The solution was incubated overnight at 4 ˚C, recovered by centrifugation, and resuspended in 1X phosphate-buffered saline (PBS). The GII.4 HOV pTP663 Jun-Fos phage display library inserts from the sheared pKS-SagaFpA50 plasmid encoding GII.4 HOV ORF1 to ORF3 was prepared for this study using the same methods as described for the GI.1 pTP663 Jun-Fos phage display library.

### Affinity selection

The first round of affinity selection was performed by placing a 100 μl (1 mg) of Pierce Protein A/G Magnetic beads (Thermo Scientific, #88803) into a 1.5 ml microcentrifuge tube and washing with 1X TBS-T with 0.05% Tween-20. Then, 500 μl of human sera (diluted 1:50) was immobilized on the beads in 1X TBS at room temperature for 1 h. The rabbit anti-GI.1 VLP antibody sera was immobilized on beads following the same procedure. The HJT-R3-A9 scFv antibody was purified previously after protein expression in *E. coli*^25,93^. The antibody was immobilized at 200 μg/ml in 1X TBS on the beads at room temperature for 1 h. For all samples, after washing with 1 ml 1X TBS-T with 0.05% Tween-20, the tubes were placed on a magnetic stand where the magnetic beads were collected, and the supernatant discarded. The beads with immobilized human sera, rabbit sera, and the HJT-R3-A9 antibody were then washed three times with 1 ml of 1X TBS-T with 0.05% Tween-20 and blocked at room temperature for 1 h with 1 ml of 1X TBS-T with 0.05% Tween-20 and 0.5% BSA. The beads were washed three times with 1X TBS-T with 0.05% Tween-20, and 5 × 10^11^ library phages were added per tube in 1X TBS and incubated at room temperature for 2 h. The beads were then washed 3 times with 1X TBS-T with 0.05% Tween-20, and phages were eluted with 500 μl per sample of 0.2 M glycine (pH 2.2), 1 mg/ml bovine serum albumin (BSA) elution buffer and neutralized with 100 μl of 2 M Tris (pH 8.5) per tube. An aliquot of the eluted phages (250 μl) was used to infect 1 ml of *E. coli* XL1-Blue cells, this mixture was inoculated in 50 ml 2YT medium containing 100 μg/ml ampicillin, and a total of 10^10^ phages/ml, or 100 μl, of M13KO7 helper phage (New England BioLabs, #N0315S), and the culture were incubated overnight at 37 ˚C to amplify the phages. Amplified phages were precipitated using PEG8000/2.5 M NaCl and collected by centrifugation at 4 kg for 30 min. Then the phages were resuspended in 1X PBS and a small aliquot was used to determine the phage titer. This phage preparation was then used for the next round of affinity selection.

### Next-Generation DNA Sequencing

Amplicon-based NGS with the Illumina platform was performed to determine the DNA sequences of inserts in the naive library and after each round of affinity selection^25^. PCR amplification was performed using the amplified phage of the naive library and the enriched libraries as the template. The PCR primers contained a 7-bp barcode sequence at the 5′ end. The barcode sequence is unique to each PCR mixture and has the purpose of assigning sequences to each experiment. After agarose gel electrophoresis and gel extraction, the DNA was extracted, purified using a Qiagen gel purification kit, pooled and used for Illumina NGS.

Custom Perl scripts were used on the deep sequencing fastq files to sort the reads, or inserts, into their respective experiments based on the barcodes associated with the amplicons. Another custom Perl script was then used to align the insert sequences to the reference genome to determine the identity and number of occurrences of each insert in each enriched library and the naive library. The Gnuplot program was used to plot the 5′ end positions, 3′ end positions, and number of occurrences of each insert on the x, y, and z axes, respectively. The inserts with a count of 5 or above were selected for the coverage score determination. Coverage scores were determined using the bedtools genomecov program and were defined as the per-nucleotide number of occurrences for each insert after alignment to the reference genome: pKS-NV68 plasmid for the GI.1 experiments and pKS-SagaFpA50 plasmid for the GII.4 HOV experiments^27,28,94^. The inserts that are in-frame to GI.1 and GII.4 HOV ORF1 to ORF3 in the pKS-NV68 and pKS-SagaFpA50 plasmids were determined and counted. The GI.1 and GII.4 HOV ORF2 were divided into consecutive 8-mer long windows. The ratio of in-frame inserts in a window for the naive library and the libraries after affinity selections was defined as number of occurrences of the in-frame inserts divided by the total number of inserts in each window. The ratio of all the in-frame inserts in the naive library and after each round of affinity selection for every individual was calculated by dividing the number of in-frame inserts by the total number of inserts in the experiment.

### Dendrogram construction

A custom Python script was used to create the blocks in the dendrogram. Seaborn, a statistical data visualization library based on matplotlib, was used to cluster the blocks with necessary semantic mapping and statistical aggregation^95^.

### Reporting summary

Further information on research design is available in the Nature Research Reporting Summary linked to this article.

## Supplementary information

Supplemental figures

REPORTING SUMMARY

## Data Availability

The datasets generated during and/or analyzed during the current study are available from the corresponding author on reasonable request.

## Code availability

Custom Perl scripts were used to process the deep sequencing data and are available at Github link: https://github.com/Palzkill-Lab/Norovirus_epitopes.
