# Supplementary material for: Mapping human norovirus antigens during infection reveals the breadth of the humoral immune response
Source: NPJ Vaccines. 2023 Jun 6;8:87. doi: 10.1038/s41541-023-00683-1 (PMC10242225; doi:10.1038/s41541-023-00683-1)
Supplement: Supplementary file 1 — Supplemental figures [file 41541_2023_683_MOESM1_ESM.pdf]

Mapping human norovirus antigens during infection reveals the breadth of the humoral immune response

Lynn Su, Wanzhi Huang, Frederick H. Neill, Mary K. Estes, Robert L. Atmar and Timothy Palzkill

Supplementary Figure 1. Schematic of HuNoV genomic phage library construction.

Supplementary Figure 2. Deep sequencing analysis of the inserts present in the NoV Jun-Fos library before and after affinity selection versus day 14-30 post-infection sera.

Supplementary Figure 3. In-frame fraction analysis of NoV Jun-Fos library versus post-infection sera before and after affinity selection.

Supplementary Figure 4. Alignment of peptides with high coverage after two rounds of affinity selection with sera of subject 715.

Supplementary Figure 5. Deep sequencing analysis of the inserts present in the GII.4 HOV Jun-Fos library before and after affinity selection versus GII.4 antisera.

Supplementary Figure 6. In-frame fraction analysis of GII.4 HOV Jun-Fos library before and after affinity selection versus GII.4 antisera.

Supplementary Figure 7. Deep sequencing analysis of the inserts present in the GII.4 HOV Jun-Fos library before and after affinity selection versus GII.4 antisera.

Supplementary Figure 8. Dendrogram of GII.4 HOV epitope profiles.

Supplementary Figure 9. Alignment of epitope sequences of GI.1 Norwalk to that of GII.4 HOV.

Supplementary Table 1. List of epitopes from all the sera identified after two rounds of affinity selections with the NoV Jun-Fos library.

Supplementary Table 2. List of epitopes from all the sera identified after two rounds of affinity selections with the GII.4 HOV Jun-Fos library.

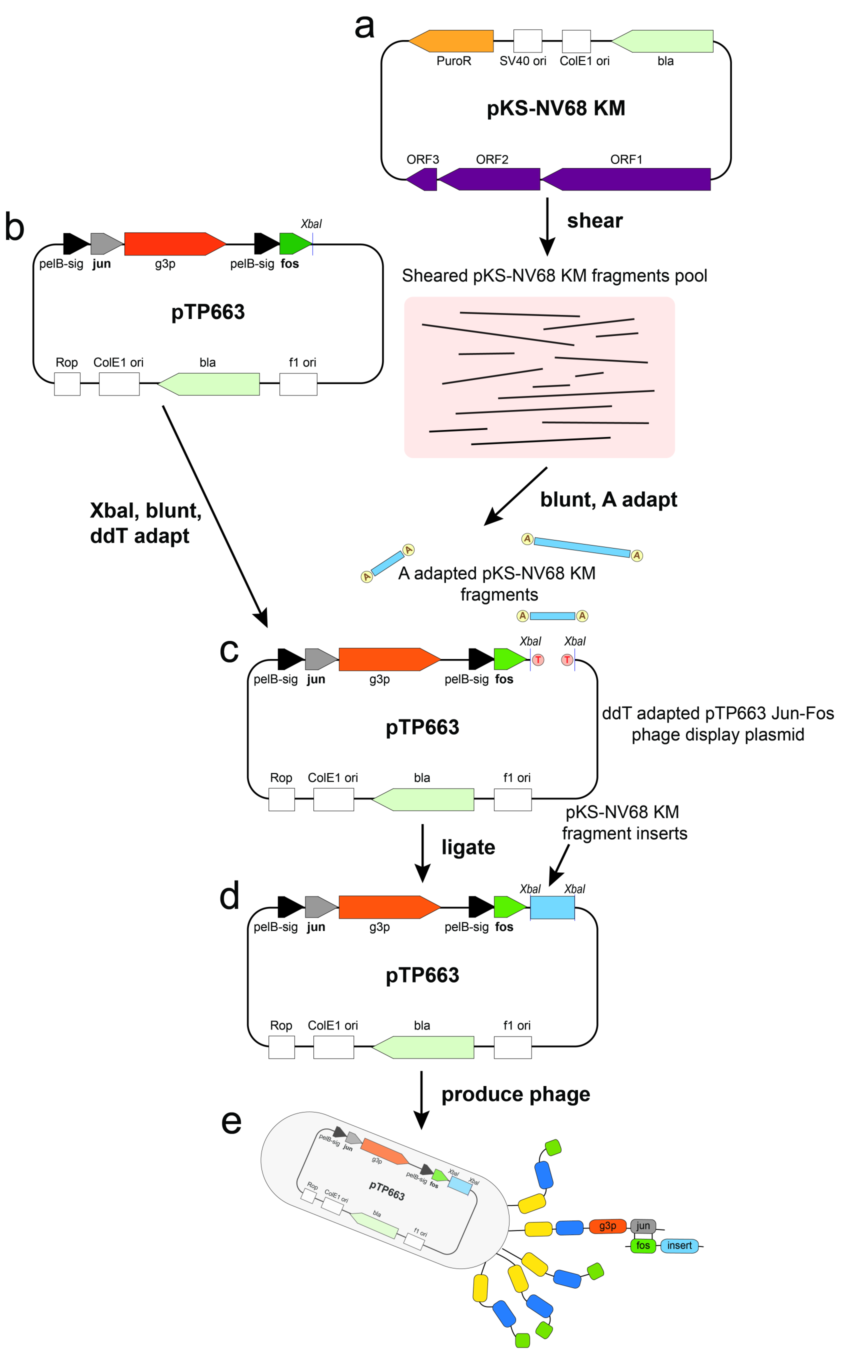

Supplementary Figure 1. Schematic of HuNoV genomic phage library construction. **a** Schematic illustration of pKS-NV68 KM plasmid, which contains the GI.1 open reading frames (ORFs) as indicated in purple, that was sheared to construct the HuNoV phage library. Since the entire plasmid was sheared, the library contains inserts from all regions of the plasmid in addition to ORFs 1 to 3. **b** Schematic illustration of the pTP663 Jun-Fos phage display plasmid. **c** Sheared DNA of 100 to 500 bp was adapted with A. **d** The pTP663 plasmid was digested with XbaI, the ends were adapted with T, and the fragments were ligated with the A-adapted sheared DNA to form the library. **e** The library clones were transformed into *E. coli*, pooled, and helper phage was added to propagate phages for the phage library to be used for affinity selection.

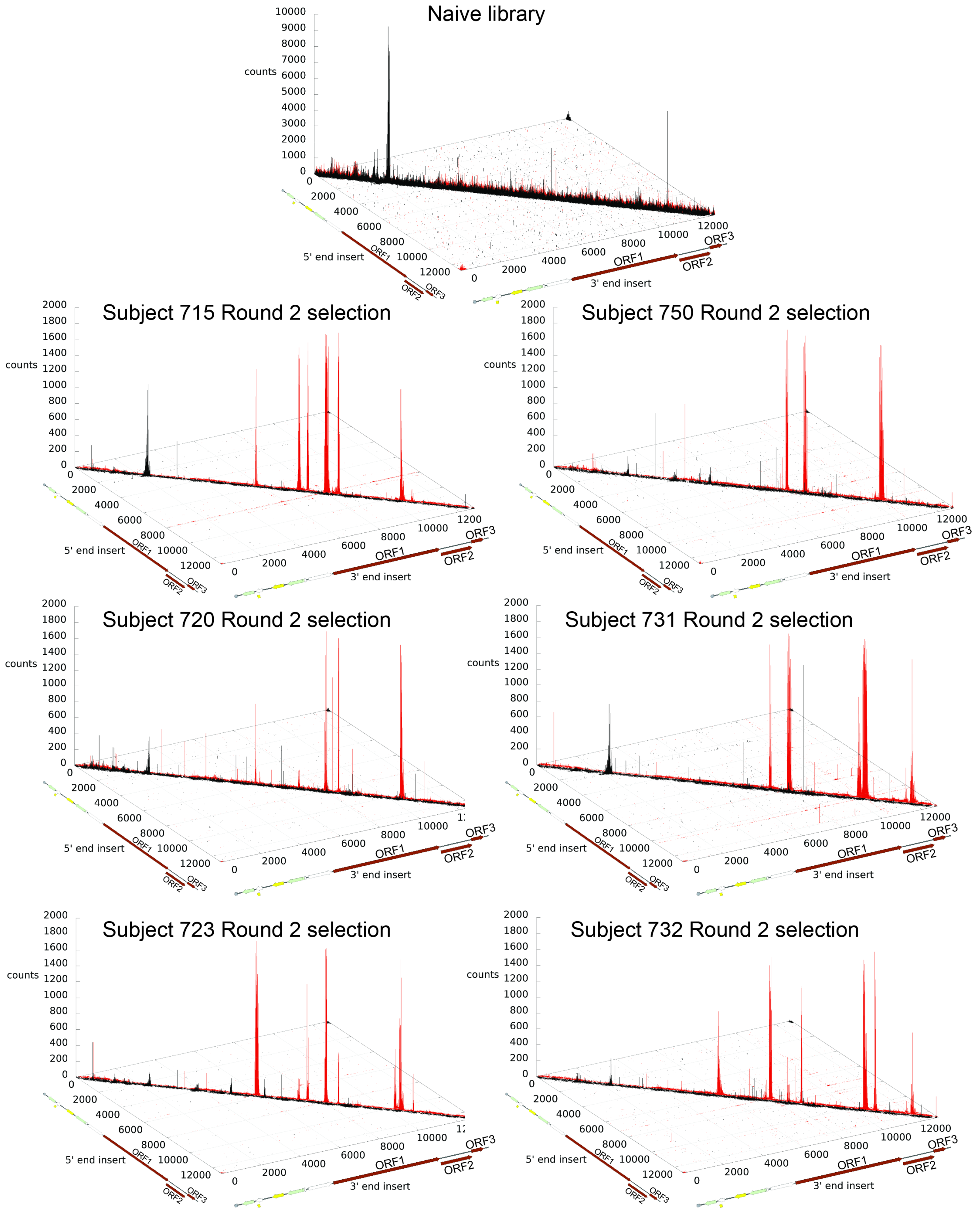

Supplementary Figure 2. Deep sequencing analysis of the inserts present in the NoV Jun-Fos library before and after affinity selection versus day 14-30 post-infection sera. Distribution of inserts after two rounds of affinity selection with sera of subjects 715, 720, 723, 750, 731, and 732.

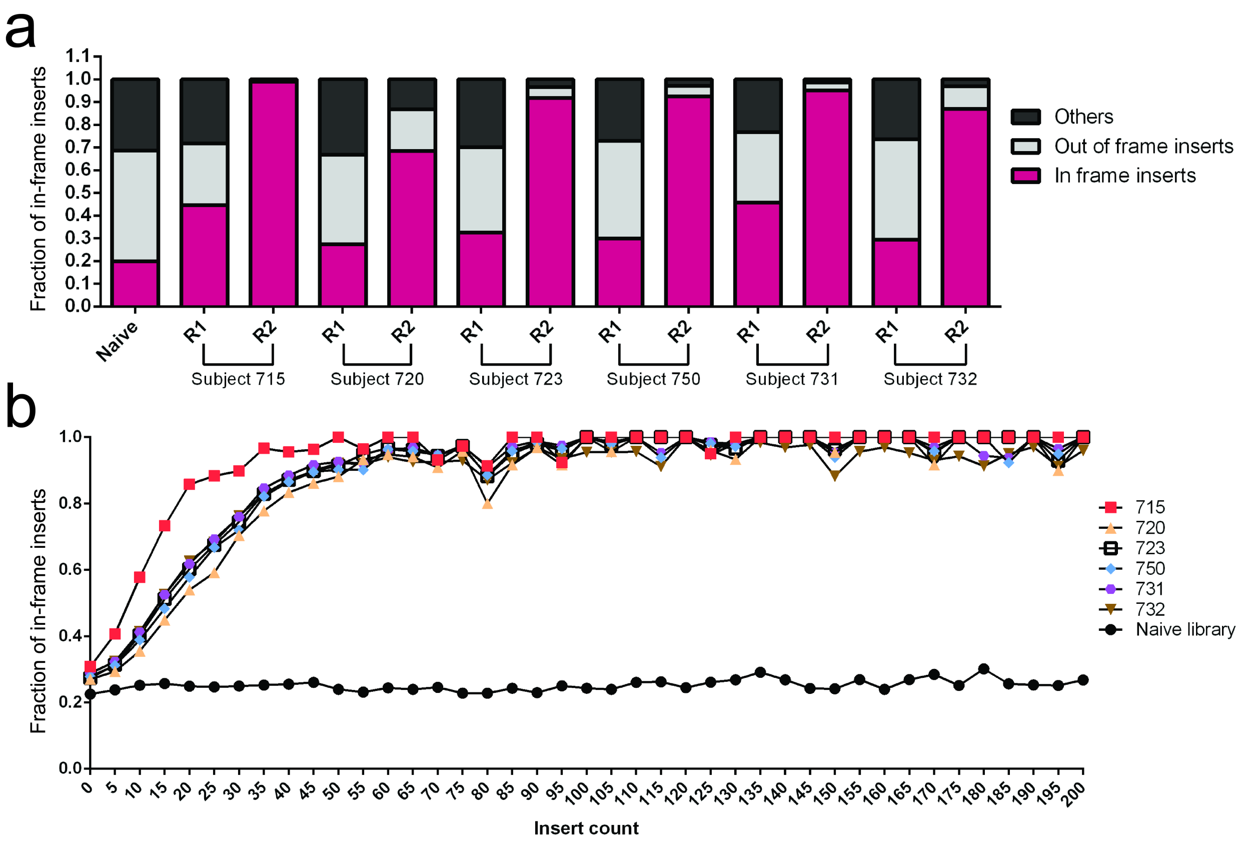

Supplementary Figure 3. In-frame fraction analysis of NoV Jun-Fos library versus post-infection sera before and after affinity selection. **a** The fraction of all the in-frame inserts in the naïve library and each round of affinity selection with subjects 715, 720, 723, 750, 731, and 732 sera. The fraction of the in-frame inserts is shown on the y-axis while the naïve library and the individual sera names are shown on the x-axis. The fraction of the inserts that are in-frame to the HuNoV ORFs are shown in magenta while the fraction of the out-of-frame inserts and the inserts that are not within HuNoV ORFs are shown in gray and black, respectively. The fraction of the in-frame inserts was determined by dividing the number of in-frame inserts by the total number of inserts in that experiment. **b** The fraction of in-frame reads for each insert count group in the naïve library and the libraries after the second round of affinity selection. The fraction of in-frame inserts is shown on the y-axis while the insert count groups are shown on the x-axis. The fraction of the in-frame inserts per insert count group is determined by dividing the number of in-frame inserts by the total number of inserts in that insert count group.

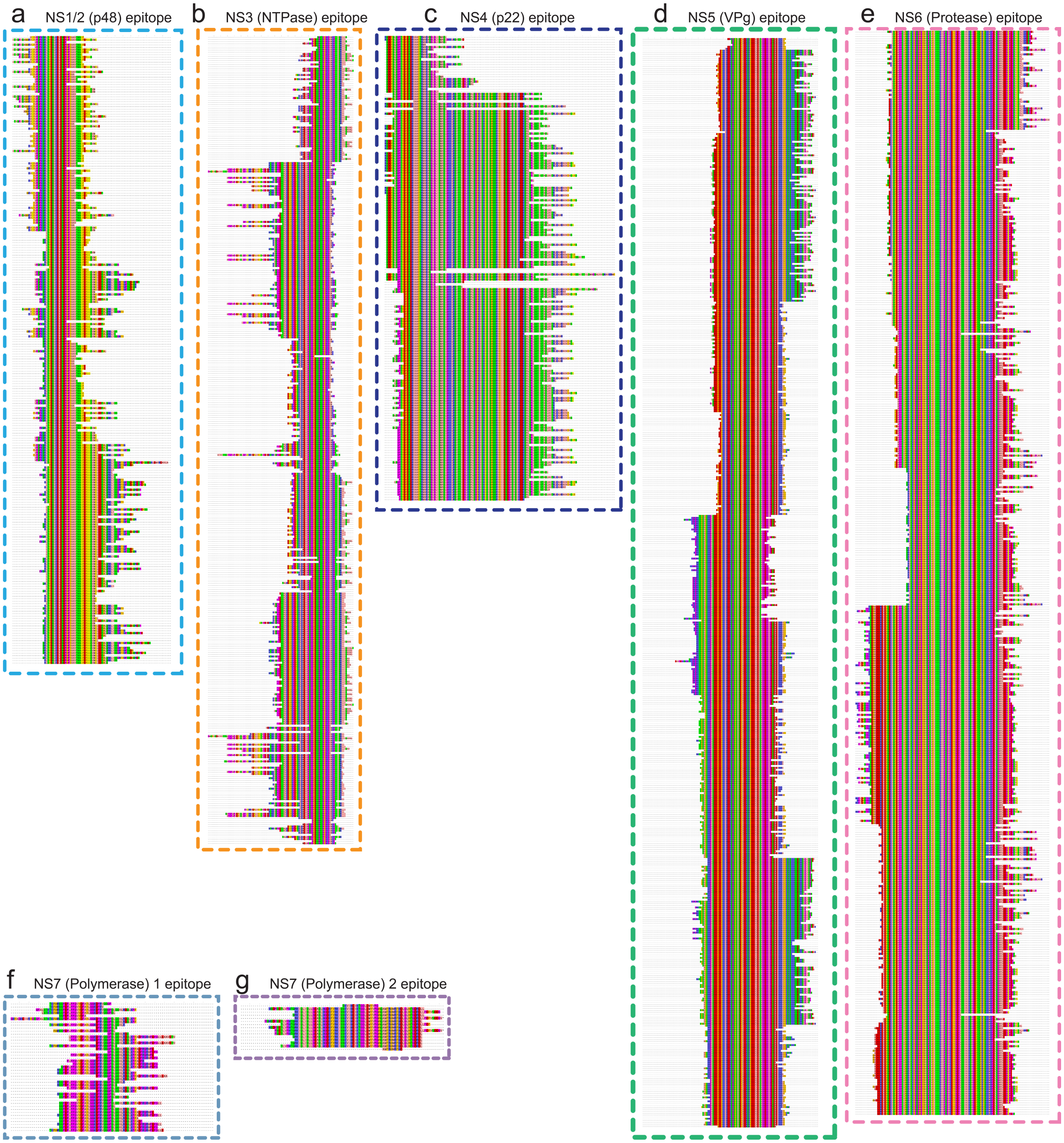

Supplementary Figure 4. Alignment of peptides with high coverage after two rounds of affinity selection with sera of subject 715. The numbering at the top indicates the amino acid residue positions in the nonstructural proteins. The Jalview Zappo color scheme is used, in which aliphatic/hydrophobic residues (I, L, V, A, and M) are peach, aromatic residues (F, W, and Y) are gold, positively charged residues (K, R, and H) are blue, negatively charged residues (D and E) are red, hydrophilic residues (S, T, N, and Q) are green, conformationally special residues (G and P) are magenta, and cysteine is yellow. The peptide alignments define the anti-GI.1 nonstructural protein epitopes in **a** NS1/2 (p48), **b** NS3 (NTPase), **c** NS4 (p22), **d** NS5 (VPg), **e** NS6 (protease), and **(f, g)** NS7 (RdRp).

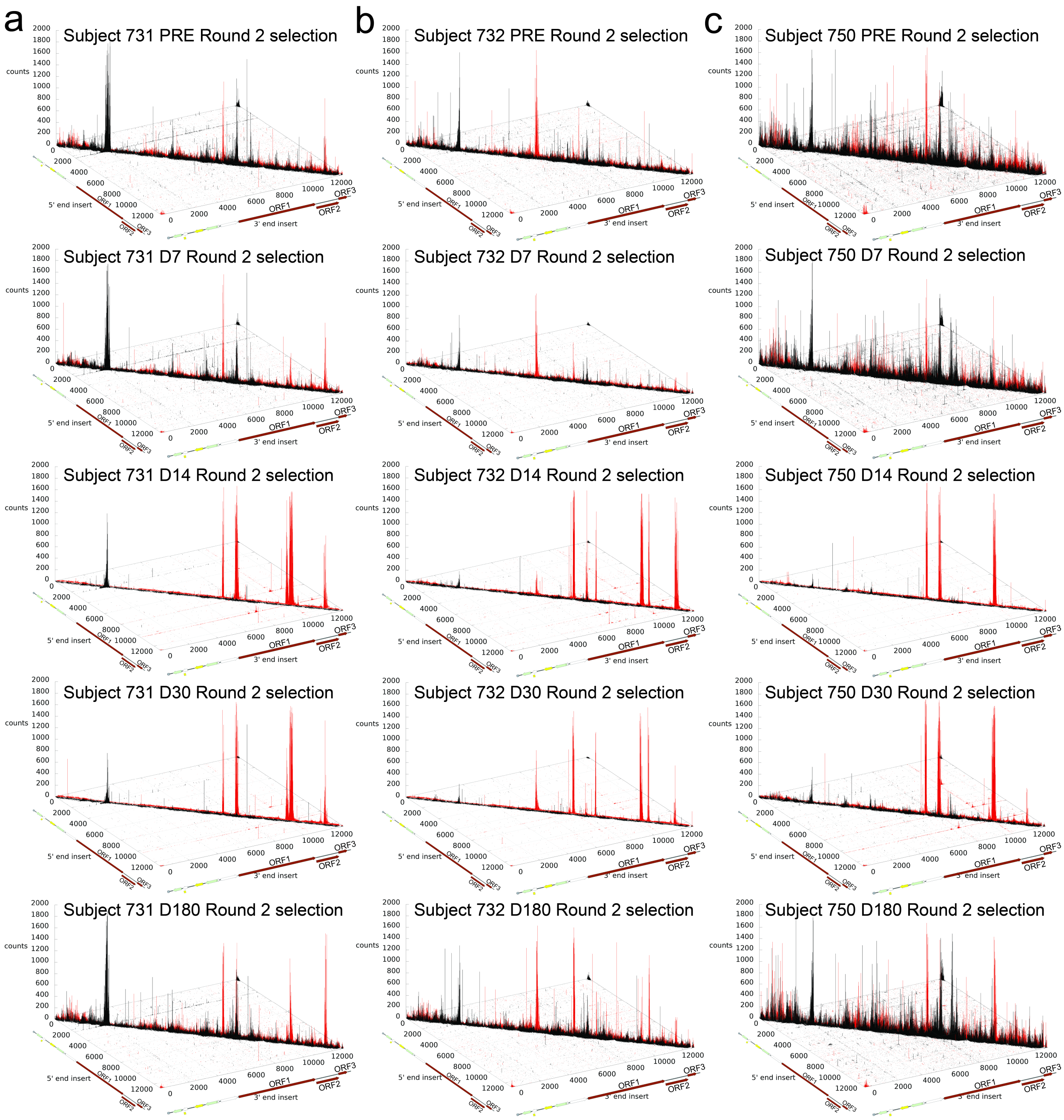

Supplementary Figure 5. Deep sequencing analysis of the inserts present in the HuNoV Jun-Fos library before and after affinity selection versus pre- and post-infection sera. **a** Distribution of inserts after two rounds of affinity selection with subject 731 sera before challenge, 7 days after challenge, 14 days after challenge, 30 days after challenge, and 180 days after challenge. **b** The distribution of inserts after two rounds of affinity selection with subject 732 sera before challenge, 7 days after challenge, 14 days after challenge, 30 days after challenge, and 180 days after challenge. **c** The distribution of inserts after round 2 of affinity selection with subject 750 sera before challenge, 7 days after challenge, 14 days after challenge, 30 days after challenge, and 180 days after challenge.

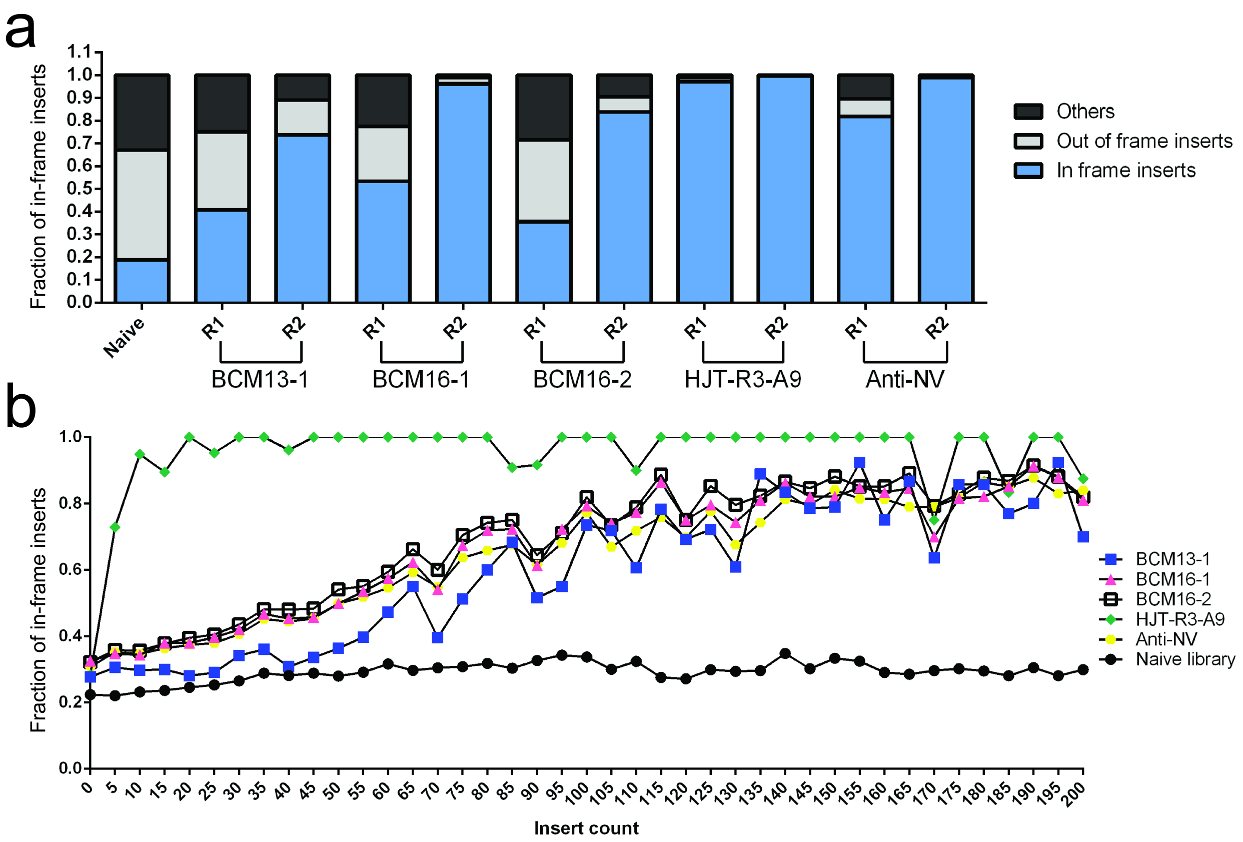

Supplementary Figure 6. In-frame fraction analysis of GII.4 HOV Jun-Fos library before and after affinity selection versus GII.4 antisera. **a** The fraction of all the in-frame inserts in the naïve library and each round of affinity selection with subjects BCM16-1, BCM13-1, and BCM16-2 sera, HJT-R3-A9 antibody, and Anti-NV. The fraction of the in-frame inserts is shown on the y-axis while the naïve library and the individual sera names are shown on the x-axis. The fraction of the inserts that are in-frame to the HuNoV ORFs are shown in blue while the fraction of the out-of-frame inserts and the inserts that are not within HuNoV ORFs are shown in gray and black, respectively. The fraction of the in-frame inserts is determined by dividing the number of in-frame inserts by the total number of inserts in that experiment. **b** The fraction of in-frame reads for each insert count group in the naïve library and the libraries after affinity selections. The fraction of in-frame inserts is shown on the y-axis while the insert count groups are shown on the x-axis. The fraction of the in-frame inserts per insert count group is determined by dividing the number of in-frame inserts by the total number of inserts in that insert count group.

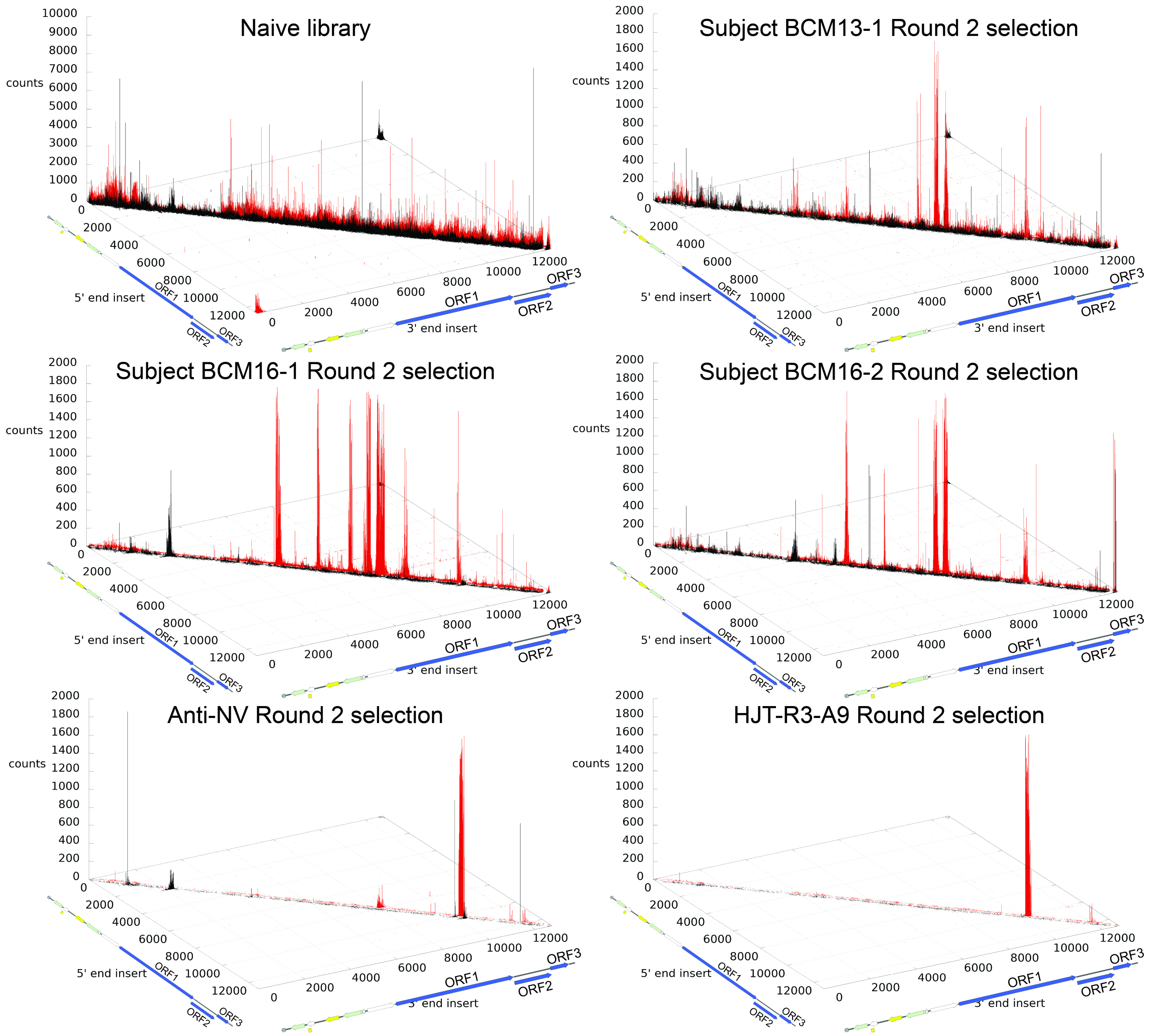

Supplementary Figure 7. Deep sequencing analysis of the inserts present in the GII.4 HOV Jun-Fos library before and after affinity selection versus GII.4 antisera. Distribution of inserts after two rounds of affinity selection with BCM16-1 sera, BCM13-1 sera, BCM16-2 sera, anti-NV rabbit polyclonal antibodies, HJT-R3-A9 scFv antibody.

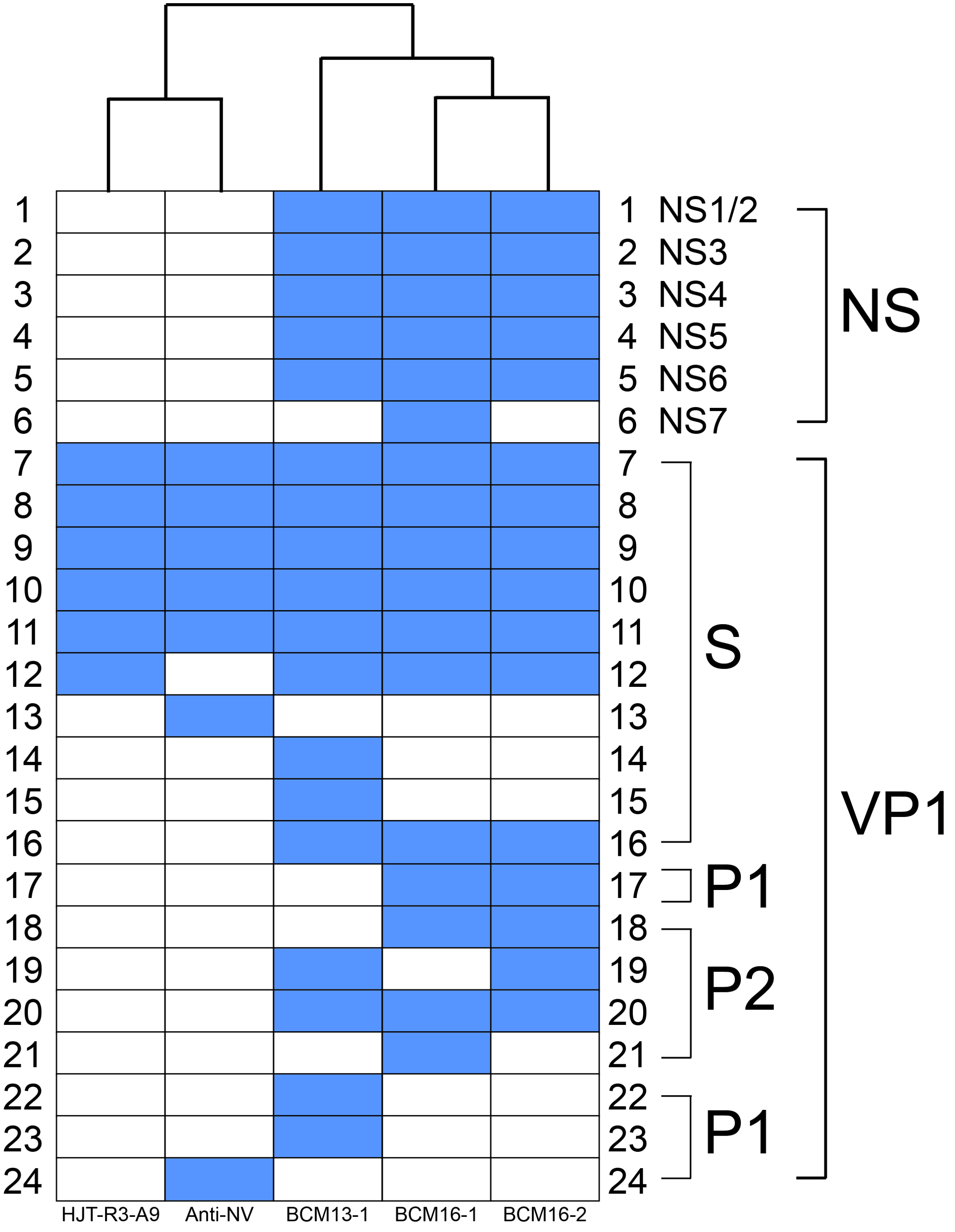

Supplementary Figure 8. Dendrogram of GII.4 HOV epitope profiles. A dendrogram comparing the epitope profiles shared among the four sera and HJT-R3-A9 scFv antibody. Blue blocks indicate the epitope that an individual has in the specified nonstructural or structural protein domain.

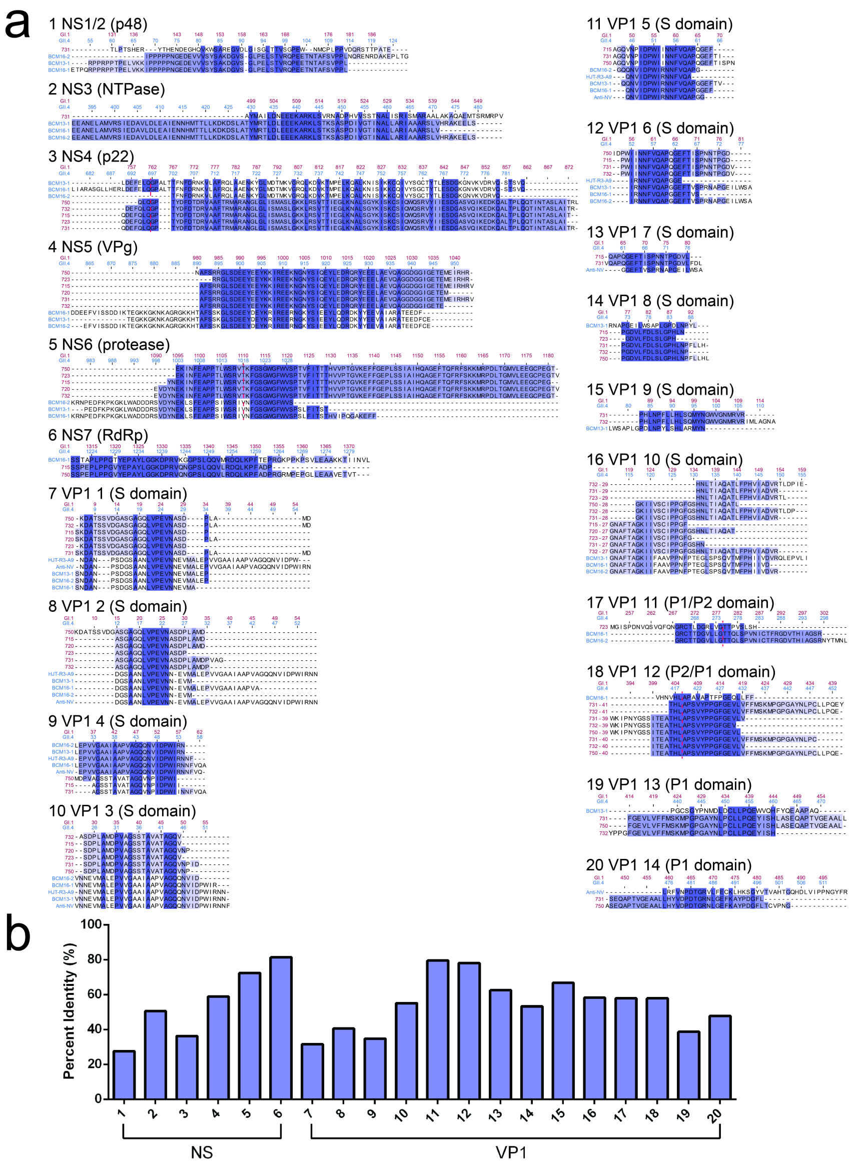

Supplementary Figure 9. Alignment of epitope sequences of GI.1 Norwalk to that of GII.4 HOV. **a** Jalview Percent Identity (PID) is used to align sequences from GI.1 Norwalk and GII.4 HOV and visualize the percent identity the sequences share. The numbering at the top indicates the amino acid residue positions in the corresponding nonstructural and structural proteins. GI.1 Norwalk positions are shown in red while the GII.4 HOV positions are shown in blue. The leftmost column indicates the name of the sera or antibody while the different shades of purple indicate the percent identity in the alignments. Percent identity is calculated according to the percentage of the residues in each column that match the consensus sequence. The darkest shade purple indicates 80% conservation between the GI.1 Norwalk and GII.4 HOV sequences. The next lighter grade of purple indicates 60%, and 40% and 20% for the subsequently lighter shades. **b** The NCBI Multiple Sequence Alignment Viewer (MSA) is used to calculate the percent identity between the GI.1 Norwalk and GII.4 HOV epitope sequences. Percent identity is calculated as the number of matches in an alignment row relative to the alignment length, where the alignment length is the aligned sequence minus any gaps for all other alignments.

		715 epitope sequence	715 epitope position	720 epitope sequence	720 epitope position	723 epitope sequence	723 epitope position	731 epitope sequence	731 epitope position	732 epitope sequence	732 epitope position	750 epitope sequence	750 epitope position	shared epitope sequence	shared epitope position	epitope length	shared among
1	1-p48									TLPTSHERYTHENDEGHQVKWSAREGVDLGISGLTTVSGPEWNMCPLPPVDQRSTTPATE	131 190			TLPTSHERYTHENDEGHQVKWSAREGVDLGISGLTTVSGPEWNMCPLPPVDQRSTTPATE	131 190	60	1
2	2-p48	VPQPKQRLTYDQLKELENEPWPYAAVTNNCFEFCCQVMCLEDT	245 287	PKQRLTYDQLKELENEPWPYAAVTNNCFEFCCQVM	248 282	VCYVPQPKQRLTYDQLKELENEPWPYAAVTNNCFEFCCQVMCLEDTWLQRKLIS	242 295							PKQRLTYDQLKELENEPWPYAAVTNNCFEFCCQVM	248 282	35	3
3	3-p48							QDSKLEMVRDAVLAAINGLVSRPFKDLLGKLKPLNVLNLLS	315 355					QDSKLEMVRDAVLAAINGLVSRPFKDLLGKLKPLNVLNLLS	315 355	41	1
4	1-NTPase							DLVPIVLGGIGLAIGFTRDKVSKMMKNAVDGLRAATQLGQYGL	406 448					DLVPIVLGGIGLAIGFTRDKVSKMMKNAVDGLRAATQLGQYGL	406 448	43	1
5	2-NTPase							AYMAILDNEEEKARKLSVRNADPHVVSSTNALISRISMARAALAKAQAEMTSRMRPV	498 554					AYMAILDNEEEKARKLSVRNADPHVVSSTNALISRISMARAALAKAQAEMTSRMRPV	498 554	57	1
6	3-NTPase	DTTALKDCFKPDFSHLKMELAPQGGFDNQGNTPFGKGVMKPTTINRLL	697 744	DTTALKDCFKPDFSHLKMELAPQGGFDNQGNTPFGKGVMKPTTINRL	697 743	GDTTALKDCFKPDFSHLKMELAPQGGFDNQGNTPFGKGVMKPTTINRL	696 743							DTTALKDCFKPDFSHLKMELAPQGGFDNQGNTPFGKGVMKPTTINRL	697 743	47	3
7	1-p22	QDEFQLQGPTYDFDTDRVAAFTRMARANGLGLISMASLGKKLRSVTTIEGLKNALSGYKISKCSIQWQSRVYIIESDGASVQIKEDKQALTPLQQTINTASLAIT	755 859			QDEFQLQGPTYDFDTDRVAAFTRMARANGLGLISMASLGKKLRSVTTIEGLKNALSGYKISKCSIQWQSRVYIIESDGASVQIKEDKQALTPLQQTINTASLAI	755 858	QDEFQLQGPTYDFDTDRVAAFTRMARANGLGLISMASLGKKLRSVTTIEGLKNALSGYKISKCSIQWQSRVYIIESDGASVQIKEDKQALTPLQQTINTASLAITR	755 860	DEFQLQGPTYDFDTDRVAAFTRMARANGLGLISMASLGKKLRSVTTIEGLKNALSGYKISKCSIQWQSRVYIIESDGASVQIKEDKQALTPLQQTINTASLAITR	756 860	QLQGPTYDFDTDRVAAFTRMARANGLGLISMASLGKKLRSVTTIEGLKNALSGYKISKCSIQWQSRVYIIESDGASVQIKEDKQALTPLQQTINTASLAITRL	759 861	QLQGPTYDFDTDRVAAFTRMARANGLGLISMASLGKKLRSVTTIEGLKNALSGYKISKCSIQWQSRVYIIESDGASVQIKEDKQALTPLQQTINTASLAI	759 858	100	5
8	1-VPg	AFSRRGLSDEEYEEYKKIREEKNGNYSIQEYLEDRQRYEEELAEVQAGGDGGIGETEMEIRHRV	981 1044	AFSRRGLSDEEYEEYKKIREEKNGNYSIQEYLEDRQRYEEELAEVQAGGDGGIGETEM	981 1044	RRGLSDEEYEEYKKIREEKNGNYSIQEYLEDRQRYEEELAEVQAGGDGGIGETEMEIRHR	984 1043	AFSRRGLSDEEYEEYKKIREEKNGNYSIQEYLEDRQRYEEELAEVQAGGDGGIGETEMEIRHRV	981 1044	AFSRRGLSDEEYEEYKKIREEKNGNYSIQEYLEDRQRYEEELAEVQAGGDGGIGETE	981 1037	NAFSRRGLSDEEYEEYKKIREEKNGNYSIQEYLEDRQRYEEELAEVQAGGDGGIGETEMEIRHR	980 1043	RRGLSDEEYEEYKKIREEKNGNYSIQEYLEDRQRYEEELAEVQAGGDGGIGETE	984 1037	54	6
9	1-protease	YNEKINFEAPPTLWSRVTKFGSGWGFWVSPTVFITTTHVVPTGVKEFFGEPLSSIAIHQAGEFTQFRFSKKMRPDLTGMVLEEGCPEG	1093 1180	EVDYNEKINFEAPPTLWSRVTKFGSGWGFWVSPTVFITTTHVVPTGVKEFFGEPLSSIAIHQAGEFTQFRFSKKMRPDLTGMVLEEGCPEGTV	1090 1186	EKINFEAPPTLWSRVTKFGSGWGFWVSPTVFITTTHVVPTGVKEFFGEPLSSIAIHQAGEFTQFRFSKKMRPDLTGMVLEEGCPEGTV	1095 1182			EVDYNEKINFEAPPTLWSRVTKFGSGWGFWVSPTVFITTTHVVPTGVKEFFGEPLSSIAIHQAGEFTQFRFSKKMRPDLTGMVLEEGCPEGT	1090 1181	EKINFEAPPTLWSRVTKFGSGWGFWVSPTVFITTTHVVPTGVKEFFGEPLSSIAIHQAGEFTQFRFSKKMRPDLTGMVLEEGCPEGT	1095 1181	EKINFEAPPTLWSRVTKFGSGWGFWVSPTVFITTTHVVPTGVKEFFGEPLSSIAIHQAGEFTQFRFSKKMRPDLTGMVLEEGCPEG	1095 1180	86	5
10	1-RdRp	SSPEPLPPGVYEPAYLGGKDPRVQNGPSLQQVLRDQLKPFADP	1311 1353									SSPEPLPPGVYEPAYLGGKDPRVQNGPSLQQVLRDQLKPFADPRGRMPEPGLLEAAVETVT	1311 1371	SSPEPLPPGVYEPAYLGGKDPRVQNGPSLQQVLRDQLKPFADP	1311 1353	43	2
11	2-RdRp							RLLWGADLGTVVRAARAFGPFCDAIKSHVIKLPIKVGMNTIEDGPLIYAEHAKYKNHFDADYTAWDSTQNRQIMTESF	1462 1539	SHVIKLPIKVGMNTIEDGPLIYAEHAKYKNHFDADYTAWDSTQNRQIMTESF	1488 1539			SHVIKLPIKVGMNTIEDGPLIYAEHAKYKNHFDADYTAWDSTQNRQIMTESF	1488 1539	52	2
12	3-RdRp							GLPSGFPCTSQVNSINHWIITLCALSEATGLSPDVVQSMSYFSFYGDDEIVSTDIDFDPARLTQILKEYGLKPTRPD	1577 1653					GLPSGFPCTSQVNSINHWIITLCALSEATGLSPDVVQSMSYFSFYGDDEIVSTDIDFDPARLTQILKEYGLKPTRPD	1577 1653	77	1
13	4-RdRp	TLVPHTQRKIQLISLLGEASLHGEKFYRKISSKVIHEIKTGGLEMYVPGWQAMFRWMRFHDLGLWTGDRDLL	1709 1780			PSETLVPHTQRKIQLISLLGEASLHGEKFYRKISSKVIHEIKTGGLEMYVPGWQAMFRWMRFHDLGLWTGDRDLLPE	1706 1782	SDPSETLVPHTQRKIQLISLLGEASLHGEKFYRKISSKVIHEIKTGGLEMYVPGWQAMFRWMRFHDLGLWTGDRDLLPEFVNDD	1704 1787			HSDPSETLVPHTQRKIQLISLLGEASLHGEKFYRKISSKVIHEIKTGGLEMYVPGWQAMFRWMRFHDLGLWTGDRDLLPEF	1703 1783	TLVPHTQRKIQLISLLGEASLHGEKFYRKISSKVIHEIKTGGLEMYVPGWQAMFRWMRFHDLGLWTGDRDLL	1709 1780	72	4
14	1-VP1 (S domain)	SKDATSSVDGASGAGQLVPEVNASDP	5 30	SKDATSSVDGASGAGQLVPEVNASDPLA	5 32	KDATSSVDGASGAGQLVPEVNASD	6 29	SKDATSSVDGASGAGQLVPEVNASDPLAMD	5 34	KDATSSVDGASGAGQLVPEVNASDPLAMD	6 34	KDATSSVDGASGAGQLVPEVNASDPLAMD	6 34	KDATSSVDGASGAGQLVPEVNASD	6 29	24	6
15	1-VP1 (S domain)	ASGAGQLVPEVNASDPLAMD	15 34	ASGAGQLVPEVNASDPLAMD	15 34	ASGAGQLVPEVNASDP	15 30	ASGAGQLVPEVNASDPLAMDPVAG	15 38	ASGAGQLVPEVNASDPLAMDP	15 35	ASGAGQLVPEVNASDPLAMDPVAG	15 38	ASGAGQLVPEVNASDP	15 30	16	6
16	1-VP1 (S domain)	PEVNASDPLAMDPVA	23 36	PEVNASDPLAMDPVAG	23 38	GQLVPEVNASDPLAMDPVA	19 37	GQLVPEVNASDPLAMDPVAGSSTAVAT	19 45	GQLVPEVNASDPLAMDPVAGS	19 39	VPEVNASDPLAMDPVAGS	22 39	PEVNASDPLAMDPVA	23 37	15	6
17	1-VP1 (S domain)	SDPLAMDPVAGSSTAVATAGQV	28 49	SDPLAMDPVAGSSTAVATAGQVNP	28 51	SDPLAMDPVAGSSTAVATAGQV	28 49	SDPLAMDPVAGSSTAVATAGQVNPID	28 53	ASDPLAMDPVAGSSTAVATAGQV	27 49	SDPLAMDPVAGSSTAVATAGQVNP	28 51	SDPLAMDPVAGSSTAVATAGQV	28 49	22	6
18	1-VP1 (S domain)	AGSSTAVATAGQVNPI DPWII	37 57					AGSSTAVATAGQVNPIDPWIINNFVQA	37 63			MDPVAGSSTAVATAGQVNPIDPWI	33 56	AGSSTAVATAGQVNPIDPWI	37 56	20	3
19	1-VP1 (S domain)	AGQVNPIDPWIINNFVQAPQGEF	46 68					AGQVNPIDPWIINNFVQAPQGEFTI	46 70			AGQVNPIDPWIINNFVQAPQGEFTISPN	46 73	AGQVNPIDPWIINNFVQAPQGEF	46 68	23	3
20	1-VP1 (S domain)	PWIINNFVQAPQGEFT ISPNNTPGD	54 78					PWIINNFVQAPQGEFTISPNNTPGDV	54 79	PWIINNFVQAPQGEFTISPNNTPGDV	54 79	IDPWIINNFVQAPQGEFTISPNNTPGD	52 78	PWIINNFVQAPQGEFTISPNNTPGD	54 78	25	4
21	1-VP1 (S domain)	QAPQGEFTISPNNTPGDVL	62 80					VQAPQGEFTISPNNTPGDVLFDL	61 83					QAPQGEFTISPNNTPGDVL	62 80	19	2
22	1-VP1 (S domain)	PGDVLFDLSLGPHLN	76 90			GDVLFDLSLGPH	77 88	PGDVLFDLSLGPHLNPFLLH	76 95	PGDVLFDLSLGPHLNP	76 91	PGDVLFDLSLGPHLNPFLLHL	76 96	GDVLFDLSLGPH	77 88	12	5
23	1-VP1 (S domain)							PHLNPFLLHLSQMYNGWVGNMRVR	87 110	PHLNPFLLHLSQMYNGWVGNMRVRIMLAGNA	87 117			PHLNPFLLHLSQMYNGWVGNMRVR	87 110	24	2
24	1-VP1 (S domain)	LHLSQMYNGWVGNMRVRIMLAGN	94 116					LHLSQMYNGWVGNMRVRIMLAGN	94 116	LHLSQMYNGWVGNMRVRIMLAGNAFTAGKIIV	94 125			LHLSQMYNGWVGNMRVRIMLAGN	94 116	23	3
25	1-VP1 (S domain)							GWVGNMRVRIMLAGNAFTAGKIIV	102 125	MYNGWVGNMRVRIMLAGNAFTAGKIIVSCIPPGFGSHNLTIAQAT	99 143	GWVGNMRVRIMLAGNAFTAGKIIVSCIPPG	102 131	GWVGNMRVRIMLAGNAFTAGKIIV	102 125	24	3
26	1-VP1 (S domain)	VRIMLAGNAFTAGKIIVSCIPPGFGSHNLTIAQATLFPHV	109 148			VRIMLAGNAFTAGKIIVSCIPPGFGSHNLTIAQAT	109 143	VRIMLAGNAFTAGKIIVSCIPPGFGSHNLTIAQAT	109 143	VRIMLAGNAFTAGKIIVSCIPPGFGSHNLTIAQATLFPHVI	109 149	VRIMLAGNAFTAGKIIVSCIPPGFGSHNLTIAQATLFPHV	109 148	VRIMLAGNAFTAGKIIVSCIPPGFGSHNLTIAQAT	109 143	35	5
27	1-VP1 (S domain)	GNAFTAGKIIVSCIPPGF	115 132	GNAFTAGKIIVSCIPPGFGSHNLTIAQAT	115 143	GNAFTAGKIIVSCIPPGFG	115 133	GNAFTAGKIIVSCIPPGFGSHN	115 136	GNAFTAGKIIVSCIPPGFGSHNLTIAQATLFPHVIADVR	115 153			GNAFTAGKIIVSCIPPGF	115 132	18	5
28	1-VP1 (S domain)							GKIIVSCIPPGFGSHNLTIAQATLFPHVIADVR	121 153	GKIIVSCIPPGFGSHNLTIAQATLFPHVIADVRTLDP	121 157	GKIIVSCIPPGFGSHNLTIAQATL	121 144	GKIIVSCIPPGFGSHNLTIAQATL	121 144	24	3
29	1-VP1 (S domain)					HNLTIAQATLFPHVIADVRTL	135 155	HNLTIAQATLFPHVIADVR	135 153	HNLTIAQATLFPHVIADVRTLDPIE	135 159			HNLTIAQATLFPHVIADVR	135 153	19	3
30	1-VP1 (S domain)	ADVRTLDPIEVPLEDVR	150 166											ADVRTLDPIEVPLEDVR	150 166	17	1
31	1-VP1 (S domain)	RTLDPIEVPLEDVRNV	153 168	RTLDPIEVPLEDVRNV	153 168	RTLDPIEVPLEDVRNV	153 168	LDPIEVPLEDVRN	155 167	VRTLDPIEVPLEDVRNV	152 168	VRTLDPIEVPLEDVRNVLFHNNDRNQQTM	152 180	LDPIEVPLEDVRN	155 167	13	6
32	1-VP1 (S domain)									RNVLFHNNDRNQQTMRLVCMLYTPLRTGG	166 194	RNVLFHNNDRNQQTMRLVCMLYTPLR	166 191	RNVLFHNNDRNQQTMRLVCMLYTPLR	166 191	26	2
33	1-VP1 (P1 domain)											FNFLFLVPPTVEQKTRPFTLPNLPLSSL	214 241	FNFLFLVPPTVEQKTRPFTLPNLPLSSL	214 241	28	1
34	1-VP1 (P1 domain)					MGISPDNVQSVQFQNGRCTLDGRLVGTTPVSLSH	253 286							MGISPDNVQSVQFQNGRCTLDGRLVGTTPVSLSH	253 286	34	1
35	1-VP1 (P2 domain)									IQANGIGSGNYVGVLSWISPPSHPS	359 383			IQANGIGSGNYVGVLSWISPPSHPS	359 383	25	1
36	1-VP1 (P2 domain)							GNYVGVLSWISPPSHPSGSQVDLWKIPNYGSSITEAT	367 403	GNYVGVLSWISPPSHPSGSQVDLWKIP	367 393	GNYVGVLSWISPPSHPSGSQVDLWKI	367 392	GNYVGVLSWISPPSHPSGSQVDLWKI	367 392	26	3
37	1-VP1 (P2 domain)									LSWISPPSHPSGSQVDLWK	373 391	SWISPPSHPSGSQVD	374 388	SWISPPSHPSGSQVD	374 388	15	2
38	1-VP1 (P2 domain)							PSHPSGSQVDLWKIPNYGSSITEATHLAPSVYP	379 411	PSHPSGSQVDLWKIPNYGSSITEAT	379 403			PSHPSGSQVDLWKIPNYGSSITEAT	379 403	25	2
39	1-VP1 (P1 domain)							WKIPNYGSSITEATHLAPSVYPPGFGEVLV	390 419	WKIPNYGSSITEATHLAPSVYPPGFGEV	390 417	WKIPNYGSSITEATHLAPSVYPPGFGEVL	390 418	WKIPNYGSSITEATHLAPSVYPPGFGEV	390 417	28	3
40	1-VP1 (P1 domain)							ITEATHLAPSVYPPGFGEVLVFFMSKMPGPGAYNLPC	399 435	ITEATHLAPSVYPPGFGEVLV	399 419	ITEATHLAPSVYPPGFGEVLVFFMSKMPGPGAYNLPCLLPQE	399 440	ITEATHLAPSVYPPGFGEVLV	399 419	21	3
41	1-VP1 (P1 domain)							THLAPSVYPPGFGEVLVFFMSKMPGPGAYNLPCLLPQEY	403 441	THLAPSVYPPGFGEVLVFFMSKMPGPGAYNLPCLLPQE	403 440			THLAPSVYPPGFGEVLVFFMSKMPGPGAYNLPCLLPQE	403 440	38	2
42	1-VP1 (P1 domain)							FGEVLVFFMSKMPGPGAYNLPCLLPQEYISHLASEQAPTVGEAALL	414 459	YPPGFGEVLVFFMSKMPGPGAYNLPCLLPQEYISH	410 444	FGEVLVFFMSKMPGPGAYNLPCLLPQEYISHLASEQAPTVGEAAL	414 452	FGEVLVFFMSKMPGPGAYNLPCLLPQEYISH	414 444	31	3
43	1-VP1 (P1 domain)							SEQAPTVGEAALLHYVDPDTGRNLGEFKAYPDGFL	447 481			ASEQAPTVGEAALLHYVDPDTGRNLGEFKAYPDGFLTCVPNG	446 487	SEQAPTVGEAALLHYVDPDTGRNLGEFKAYPDGFL	447 481	35	2
44	1-VP1 (P1 domain)											DGFLTCVPNGASSGPQQLPING	478 499	DGFLTCVPNGASSGPQQLPING	478 499	22	1
45	1-VP1 (P1 domain)			PQQLPINGVFV	492 502			GPQQLPINGVFVFVSWVSRFYQLK	491 514			QQLPINGVFVFVSWVS	493 508	QQLPINGVFV	493 502	10	3
46	1-VP1 (P1 domain)							INGVFVFVSWVSRFYQLKP	497 515			INGVFVFVSWVS	497 508	INGVFVFVSWVS	497 508	12	2
47	1-VP1 (P1 domain)			SRFYQLKPVGTASSAR	508 523			YQLKPVGTASSARG	511 524	YQLKPVGTASSARG	511 524			YQLKPVGTASSAR	511 523	13	3
48	1-VP1 (P1 domain)							GTASSARG	517 524	GTASSARGR	517 525	LKPVGTASSARG	513 524	GTASSARG	517 524	8	3

Supplementary Table 1. List of epitopes from all the sera identified after two rounds of affinity selections with the NoV Jun-Fos library. A total of 48 unique epitopes were identified. The sequences and the positions in the GI.1 Norwalk ORF1 and ORF2 genome of each epitope are included. The shared epitopes amongst the six individuals as well as the epitopes' sequences, positions in the GI.1 Norwalk genome, length, and the number of sera that contain the epitopes are also included.

		HJT-R3-A9 epitope sequence	HJT-R3-A9 epitope position	BCM131 epitope sequence	BCM131 epitope position	BCM161 epitope sequence	BCM161 epitope position	BCM162 epitope sequence	BCM161 epitope position	Anti-NV epitope sequence	Anti-NV epitope position	shared epitope sequence	shared epitope position	epitope length	shared among
1	1-p48			RPPRPPTPELVKKIPPPPPNGEDEVVVSYSAKDGVSGLPELSTVRQPEETNTAFSVPPL	55 131	ETPQRPPRPPTPELVKKIPPPPPNGEDEVVVSYSAKDGVSGLPELSTVRQPEETNTAFSVPPL	51 113	IPPPPPNGEDEVVVSYSAKDGVSGLPELSTVRQPEETNTAFSVPPLNQRENRDAKEPLTG	68 127			IPPPPPNGEDEVVVSYSAKDGVSGLPELSTVRQPEETNTAFSVPPL	68 113	46	3
2	2-NTPase			EEANELAMVRSIEDAVLDLEAIENNHMTTLLKDKDSLATYMRTLDLEEEKARKLSTKSASPDIVGTINALLARIAAARSLVHRAKEELS	395 483	EEANELAMVRSIEDAVLDLEAIENNHMTTLLKDKDSLATYMRTLDLEEEKARKLSTKSASPDIVGTINALLARIAAARSLV	395 475	EEANELAMVRSIEDAVLDLEAIENNHMTTLLKDKDSLATYMRTLDLEEEKARKLSTKSASPDIVGTINALLARIAAARSLVHRAKEELS	395 483			EEANELAMVRSIEDAVLDLEAIENNHMTTLLKDKDSLATYMRTLDLEEEKARKLSTKSASPDIVGTINALLARIAAARSLV	395 475	81	3
3	3-p22			LDEFELQGPALTTFNFDRNKVLAFRQLAAENKYGLMDTMKVGRQLKDVKTMPELKQALKNISIKKCQIVYSGCTYTLESDGKGNVKVDRVQSTSVQ	690 785	LIARASGLLHERLDEFELQGPALTTFNFDRNKVLAFRQLAAENKYGLMDTMKVGRQLKDVKTMPELKQALKNISIKKCQIVYSGCTYTLESDGKGNVKVDRVQSTSVQ	678 785	TFNFDRNKVLAFRQLAAENKYGLMDTMKVGRQLKDVKTMPELKQALKNISIKKCQIVYSGCTYTLESDGKGNVKVDRVQ	702 780			TFNFDRNKVLAFRQLAAENKYGLMDTMKVGRQLKDVKTMPELKQALKNISIKKCQIVYSGCTYTLESDGKGNVKVDRV	702 779	78	3
4	4-VPg			TEGKKGKNKAGRGKKHTAFSSKGLSDEEYDEYKRIREERNGKYSIEEYLQDRDKYYEEVAIARATEEDFCE	874 944	DDEEFVISSDDIKTEGKKGKNKAGRGKKHTAFSSKGLSDEEYDEYKRIREERNGKYSIEEYLQDRDKYYEEVAIARATEEDF	861 942	EFVISSDDIKTEGKKGKNKAGRGKKHTAFSSKGLSDEEYDEYKRIREERNGKYSIEEYLQDRDKYYEEVAIARATEEDFCE	864 944			TEGKKGKNKAGRGKKHTAFSSKGLSDEEYDEYKRIREERNGKYSIEEYLQDRDKYYEEVAIARATEEDF	872 942	69	3
5	5-protease			PEDFKPKGKLWADDDRSVDYNEKLSFEAPPSIWSRIVNFGSGWGFWVSPSLFITST	982 1037	KRNPEDFKPKGKLWADDDRSVDYNEKLSFEAPPSIWSRIVNFGSGWGFWVSPSLFITSTHVIPQGAKEFF	979 1048	KRNPEDFKPKGKLWADDDRSVDYNEKLSFEAPPSIWSRIVNFGSGWGFWVS	979 1029			PEDFKPKGKLWADDDRSVDYNEKLSFEAPPSIWSRIVNFGSGWGFWVS	982 1029	48	3
6	6-RdRp					SSTAPLPPGTYEPAYLGGKDPRVKGGPSLQQVMRDQLKPFTEPRGKPPKPSVLEAAKKTIINVL	1220 1283					SSTAPLPPGTYEPAYLGGKDPRVKGGPSLQQVMRDQLKPFTEPRGKPPKPSVLEAAKKTIINVL	1220 1283	64	1
7	1-VP1 (S domain)	NDANPSDGSAANLVPEVNNEVMALEPVVGAAIAAPVAGQQNVIDPW	6 51	SNDANPSDGSAANLVPEVNNEVMALEP	5 31	SNDANPSDGSAANLVPEVNNEVMALEP	5 31	SNDANPSDGSAANLVPEVNNEVMA	5 28	NDANPSDGSAANLVPEVNNEVMALEPVVGAAIAAPVAGQQNVIDPWIRN	6 54	NDANPSDGSAANLVPEVNNEVMALEP	6 31	26	5
8	2-VP1 (S domain)	DGSAANLVPEVNNEVMALEPVVGAAIAAPVAGQQNVIDPWIRNN	12 55	DGSAANLVPEVNNEVM	12 27	DGSAANLVPEVNNEVMALEPVVGAAIAAPVA	12 42	DGSAANLVPEVNNEVM	12 27	DGSAANLVPEVNNEVMALEPVVGAAIAAPVAGQQNVIDPWIRNN	12 55	DGSAANLVPEVNNEVM	12 27	16	5
9	3-VP1 (S domain)	NNEVMALEPVVGAAIAAPVAGQQNVIDPWIRNN	23 55	VNNEVMALEPVVGAAIAAPVAGQQNVIDPWIRNN	22 55	VNNEVMALEPVVGAAIAAPVAGQQNVIDPWIR	22 53	VNNEVMALEPVVGAAIAAPVAGQQNVID	22 49	VNNEVMALEPVVGAAIAAPVAGQQNVIDPWIRNNF	22 56	NNEVMALEPVVGAAIAAPVAGQQNVID	23 49	27	5
10	4-VP1 (S domain)	EPVVGAAIAAPVAGQQNVIDPWIRNNF	30 56	LEPVVGAAIAAPVAGQQNVIDPWIRN	29 54	LEPVVGAAIAAPVAGQQNVIDPWIRNNFVQA	29 59	LEPVVGAAIAAPVAGQQNVIDPWIRN	29 54	EPVVGAAIAAPVAGQQNVIDPWIRNNFVQ	30 58	EPVVGAAIAAPVAGQQNVIDPWIRN	30 54	25	5
11	5-VP1 (S domain)	QNVIDPWIRNNFVQA	45 59	QQNVIDPWIRNNFVQAPGGEFTV	44 66	QNVIDPWIRNNFVQAPGGEF	45 64	GQQNVIDPWIRNNFVQAPG	43 61	QNVIDPWIRNNFVQAPGG	45 62	QNVIDPWIRNNFVQA	45 69	15	5
12	6-VP1 (S domain)	IRNNFVQAPGGE	52 63	IRNNFVQAPGGEFTVSPRNAPGEILWSA	52 79	IRNNFVQAPGGEFTVS	52 67	IRNNFVQAPGGEFTVSPRNAPGEILWSA	52 79			IRNNFVQAPGGE	52 63	12	4
13	7-VP1 (S domain)									GGEFTVSPRNAPGEILWSA	61 79	GGEFTVSPRNAPGEILWSA	61 79	19	1
14	8-VP1 (S domain)			RNAPGEILWSAPLGPDLNPYL	69 89							RNAPGEILWSAPLGPDLNPYL	69 89	21	2
15	9-VP1 (S domain)			LWSAPLGPDLNPYLSHLARMYN	76 97							LWSAPLGPDLNPYLSHLARMYN	76 97	22	1
16	10-VP1 (S domain)			GNAFTAGKIIFAAVPPNFPTEGLSPSQVTMFPHIIVDVRQLEPVLI	111 156	GNAFTAGKIIFAAVPPNFPTEGLSPSQVTMFPHIIVD	111 147	GNAFTAGKIIFAAVPPNFPTEGLSPSQVTMFPHIIVDVR	111 149			GNAFTAGKIIFAAVPPNFPTEGLSPSQVTMFPHIIVD	111 147	37	4
17	11-VP1 (P1 domain)					GRCTTDGVLLGTTQLSPVNICTFRGDVTHIAGSR	264 297	GRCTTDGVLLGTTQLSPVNICTFRGDVTHIAGSRNYTMNL	264 303			GRCTTDGVLLGTTQLSPVNICTFRGDVTHIAGSR	264 297	34	2
18	12-VP1 (P2 domain)					NNYDPTEEIPAPLGTPDFVGKIQGLLTQTTKGDGSTRGHKAT	309 350	NNYDPTEEIPAPLGTPDFVGKIQGLLTQTTKGDGSTRGHK	309 348			NNYDPTEEIPAPLGTPDFVGKIQGLLTQTTKGDGSTRGHK	309 348	40	2
19	13-VP1 (P2 domain)			TQTTKGDGSTRGHKATVYTGSAP	335 357			TQTTKGDGSTRGHKATVYTGSAP	335 357			TQTTKGDGSTRGHKATVYTGSAP	335 357	23	2
20	14-VP1 (P2domain)			TKGDGSTRGHKATVYTGSAPFTPKLGS	338 364	TKGDGSTRGHKATVYTGSAPFTPKLGS	338 364	TKGDGSTRGHKATVYTGSAPFTPKLGS	338 364			TKGDGSTRGHKATVYTGSAPFTPKLGS	338 364	27	3
21	15-VP1 (P2 domain)					VHNVHLAPAVAPTFPGEQLLFF	413 434					VHNVHLAPAVAPTFPGEQLLFF	413 434	22	1
22	16-VP1 (P1 domain)			PGCSGYPNMDLDCLLPQEWVQHFYQEAAPAQ	439 469							PGCSGYPNMDLDCLLPQEWVQHFYQEAAPAQ	439 469	31	1
23	17-VP1 (P1 domain)			PNMDLDCLLPQEWVQHFYQEAAPAQSDVALLR	445 476							PNMDLDCLLPQEWVQHFYQEAAPAQSDVALLR	445 476	32	1
24	18-VP1 (P1 domain)									LRFVNPDTGRVLFECKLHKSGYVTVAHTGQHDLVIPPNGYFR	475 516	LRFVNPDTGRVLFECKLHKSGYVTVAHTGQHDLVIPPNGYFR	475 516	42	1

Supplementary Table 2. List of epitopes from all the sera identified after two rounds of affinity selections with the GII.4 HOV Jun-Fos library. A total of 24 unique epitopes were identified. The sequences and the positions in the GII.4 HOV ORF1 and ORF2 genome of each epitope are included. The shared epitopes amongst the six individuals as well as the epitopes' sequences, positions in the GII.4 HOV genome, length, and the number of sera that contain the epitopes are also included.
