# Supplementary material for: Mapping human norovirus antigens during infection reveals the breadth of the humoral immune response
Source: NPJ Vaccines. 2023 Jun 6;8:87. doi: 10.1038/s41541-023-00683-1 (PMC10242225; doi:10.1038/s41541-023-00683-1)
Supplement: Supplementary file 2 — REPORTING SUMMARY [file 41541_2023_683_MOESM2_ESM.pdf]

nature portfolio

Corresponding author(s): Timothy Palzkill

Last updated by author(s): 2023/05/19

Reporting Summary

Nature Portfolio wishes to improve the reproducibility of the work that we publish. This form provides structure for consistency and transparency in reporting. For further information on Nature Portfolio policies, see our Editorial Policies and the Editorial Policy Checklist.

Statistics

For all statistical analyses, confirm that the following items are present in the figure legend, table legend, main text, or Methods section.

n/a	Confirmed	
☐	☒	The exact sample size (*n*) for each experimental group/condition, given as a discrete number and unit of measurement
☐	☒	A statement on whether measurements were taken from distinct samples or whether the same sample was measured repeatedly
☒	☐	The statistical test(s) used AND whether they are one- or two-sided<br>*Only common tests should be described solely by name; describe more complex techniques in the Methods section.*
☒	☐	A description of all covariates tested
☒	☐	A description of any assumptions or corrections, such as tests of normality and adjustment for multiple comparisons
☒	☐	A full description of the statistical parameters including central tendency (e.g. means) or other basic estimates (e.g. regression coefficient) AND variation (e.g. standard deviation) or associated estimates of uncertainty (e.g. confidence intervals)
☒	☐	For null hypothesis testing, the test statistic (e.g. *F*, *t*, *r*) with confidence intervals, effect sizes, degrees of freedom and *P* value noted<br>*Give P values as exact values whenever suitable.*
☒	☐	For Bayesian analysis, information on the choice of priors and Markov chain Monte Carlo settings
☒	☐	For hierarchical and complex designs, identification of the appropriate level for tests and full reporting of outcomes
☒	☐	Estimates of effect sizes (e.g. Cohen's *d*, Pearson's *r*), indicating how they were calculated

Our web collection on statistics for biologists contains articles on many of the points above.

Software and code

Policy information about availability of computer code

Data collection	Illumina processing software used in data collection.
Data analysis	Publicly available Gnuplot program used for figure preparation. Custum perl scripts used for DNA sequence data processing and analysis.<br>Github link: https://github.com/Palzkill-Lab/Norovirus_epitopes

For manuscripts utilizing custom algorithms or software that are central to the research but not yet described in published literature, software must be made available to editors and reviewers. We strongly encourage code deposition in a community repository (e.g. GitHub). See the Nature Portfolio guidelines for submitting code & software for further information.

Data

Policy information about availability of data

All manuscripts must include a data availability statement. This statement should provide the following information, where applicable:

- Accession codes, unique identifiers, or web links for publicly available datasets
- A description of any restrictions on data availability
- For clinical datasets or third party data, please ensure that the statement adheres to our policy

The datasets generated during and/or analyzed during the current study are available from the corresponding author on reasonable request.

Human research participants

Policy information about studies involving human research participants and Sex and Gender in Research.

Reporting on sex and gender	The sera samples used and information regarding the samples was previously described and published in Atmar et al, Journal of Infectious Diseases 209, (2014).
Population characteristics	The sera samples used and information regarding the samples was previously described and published in Atmar et al, Journal of Infectious Diseases 209, (2014).
Recruitment	The sera samples used and information regarding the samples was previously described and published in Atmar et al, Journal of Infectious Diseases 209, (2014).
Ethics oversight	The study was approved by the Baylor College of Medicine Institutional Review Board.

Note that full information on the approval of the study protocol must also be provided in the manuscript.

Field-specific reporting

Please select the one below that is the best fit for your research. If you are not sure, read the appropriate sections before making your selection.

☒ Life sciences ☐ Behavioural & social sciences ☐ Ecological, evolutionary & environmental sciences

For a reference copy of the document with all sections, see nature.com/documents/nr-reporting-summary-flat.pdf

Life sciences study design

All studies must disclose on these points even when the disclosure is negative.

Sample size	No sample size calculations were performed. Sample size was determined by the availability of sera samples.
Data exclusions	No data was excluded.
Replication	Rabbit polyclonal antibody sera and the HJT-R3-A9 scFv were used as controls for biopanning and deep sequencing as these experiments had been done previously and published results are available. The results obtained here are consistent with published results. The experiments contain internal controls to demonstrate that affinity selections are enriching epitope sequences as described in the manuscript.
Randomization	Data randomization was not performed.
Blinding	Data blinding was not performed as information on the sera samples was already published.

Reporting for specific materials, systems and methods

We require information from authors about some types of materials, experimental systems and methods used in many studies. Here, indicate whether each material, system or method listed is relevant to your study. If you are not sure if a list item applies to your research, read the appropriate section before selecting a response.

Materials & experimental systems

n/a	Involved in the study	
☐	☒	Antibodies
☒	☐	Eukaryotic cell lines
☒	☐	Palaeontology and archaeology
☒	☐	Animals and other organisms
☒	☐	Clinical data
☒	☐	Dual use research of concern

Methods

n/a	Involved in the study	
☒	☐	ChIP-seq
☒	☐	Flow cytometry
☒	☐	MRI-based neuroimaging

Antibodies

Antibodies used	Sera was used from infected individuals
Validation	The sera containing the anti-NV proteins is the subject of the study.
